# Explainable machine learning differentiates necrotizing fasciitis and osteomyelitis via routine blood biomarkers

**DOI:** 10.1038/s41746-026-02686-3

**Published:** 2026-04-29

**Authors:** Parhat Yasin, Zubaidanmu Aizezi, Shiming Dong, Yasen Yimit, Alimujiang Yusufu, Wei Xiang, Zhoujun Zhu, Haopeng Luan, Xinghua Song

**Affiliations:** 1 Department of Spine Surgery, The Sixth Affiliated Hospital of Xinjiang Medical University, Urumqi, Xinjiang PR China; 2 Department of Spine Surgery, The First Affiliated Hospital of Xinjiang Medical University, Urumqi, Xinjiang PR China; 3 The First Affiliated Hospital of Xinjiang Medical University, Urumqi, Xinjiang PR China; 4 Xinjiang Key Laboratory of Artificial Intelligence Assisted Imaging Diagnosis, Department of Radiology, The First People’s Hospital of Kashi Prefecture, Kashi, Xinjiang PR China; 5 Department of Joint Surgery, The First Affiliated Hospital of Xinjiang Medical University, Urumqi, Xinjiang PR China; 6 Department of Orthopedic Surgery, Shandong Provincial Hospital Affiliated to Shandong First Medical University, Jinan, Shandong PR China

**Keywords:** Biomarkers, Computational biology and bioinformatics, Diseases, Health care, Medical research

## Abstract

Necrotizing fasciitis (NF) and osteomyelitis (OM) are severe, limb-threatening infections with overlapping features, making early differentiation challenging. To address this, we developed and validated an explainable machine learning model using routine blood biomarkers from a retrospective, multi-center cohort of 3415 patients (579 NF, 2836 OM). Data from a primary center were used for model development, with data from a second center serving as an independent external testing cohort. Systematic evaluation identified an optimal 10-biomarker LightGBM model that achieved outstanding discrimination on the external cohort, with an AUC of 0.926. Beyond its high accuracy, explainability analyses confirmed the model’s predictions are driven by robust, clinically relevant markers of severe inflammation and metabolic dysfunction, reinforcing its trustworthiness. The final model was deployed as a publicly accessible web tool for real-time risk stratification. This work provides a powerful, externally validated, and explainable AI framework to augment clinical judgment, with strong potential to reduce diagnostic delays and improve outcomes for these devastating infections.

## Introduction

Severe limb infections, including necrotizing fasciitis (NF) and advanced osteomyelitis (OM), posed life- and limb-threatening emergencies with significant mortality and morbidity risks. NF progressed rapidly, often leading to death or amputation within hours to days, while chronic OM frequently resulted in limb loss without prompt intervention. Delayed treatment worsened outcomes: NF patients faced 15% mortality, with 21% requiring amputation when extremities were involved^1^. In diabetic foot infections, referrals exceeding 59 days doubled the risk of major amputation or in-hospital mortality (21% vs. 10% for early referrals), with each day’s delay increasing poor prognosis odds by 0.6%^2^. Sepsis complicated 30% of nontraumatic amputations for ischemic or infected limbs^1^ and elevated 30-day post-amputation mortality 80-fold^8^, contributing to hospital mortality rates reaching 28%^4^. The economic burden was substantial, with diabetic foot ulcer-related amputations costing over $10 billion annually in the US due to prolonged hospitalizations and secondary procedures^4^. Surgical site infections after lower extremity revascularization nearly doubled six-month amputation risks^5^. Early diagnosis proved critical—timely debridement in NF significantly reduced mortality, while emerging tools like wound microbiome profiling offered precision treatment potential, possibly curbing unnecessary amputations and costs^6^.

NF and OM shared overlapping clinical presentations, including pain, swelling, and erythema, which often delayed accurate early diagnosis. Laboratory findings further complicated differentiation, as both conditions exhibited nonspecific inflammatory markers, particularly in high-risk populations like diabetic patients^7^. Traditional diagnostic approaches for NF and OM faced significant limitations. Clinical examination alone often failed to achieve early or accurate diagnoses, particularly for NF, which frequently presented with atypical and insidious symptoms. The overlap of clinical features with other soft tissue infections further complicated differentiation, leading to delayed interventions and elevated mortality rates^8,7^. While imaging modalities like X-rays, ultrasound, CT, MRI, SPECT, and PET provided diagnostic support, they introduced challenges. Advanced techniques such as MRI and PET often delayed diagnosis due to limited availability, while their high costs restricted accessibility in resource-constrained settings^8^. Invasive aspects, including ionizing radiation exposure (X-rays, CT, SPECT, PET) and contrast-related risks for patients with renal impairment, posed additional drawbacks^5^. Although MRI demonstrated high sensitivity (96.4%) and specificity (83.8%) for OM, alternatives like X-rays showed markedly lower accuracy (sensitivity: 61.9%, specificity: 78.3%). SPECT and scintigraphy exhibited variable reliability and limited availability^9^. For NF, imaging findings often lacked definitiveness, and procedural delays could prove catastrophic. Microbiological cultures, while considered the gold standard, required 24–72 h for results—a critical limitation in rapidly progressing NF cases where immediate surgery was essential to prevent mortality or tissue loss^10^. Collectively, these diagnostic hurdles—delays from imaging and cultures, high costs of advanced modalities, and invasiveness of procedures—contributed to poorer patient outcomes and increased healthcare burdens.

Blood tests such as C-reactive protein (CRP), white blood cell count (WBC), and other standard laboratory values are routinely collected for patients presenting with suspected severe infections, ensuring that data is readily accessible for ML analysis in nearly all healthcare settings^7^. Routine blood biomarkers were particularly well-suited for machine learning (ML)-based diagnostic models due to their universal availability in emergencies and acute care settings, rapid turnaround times enabling timely clinical decision-making, and cost-effectiveness compared to advanced imaging or specialized assays. These biomarkers—including white blood cell count and CRP—also captured systemic host-response patterns critical for differentiating NF from OM, addressing a high-stakes diagnostic challenge in limb-threatening infections^11^.

Advances in ML transformed infection diagnostics through predictive models for sepsis, cellulitis, and bacteremia using electronic health record (EHR) data, enabling early detection by identifying complex patterns in heterogeneous datasets^12,13^. ML techniques were applied to clinical microbiology image analysis, automating pathogen identification in blood smears and culture plates, and demonstrated utility in immunocompromised patients for sepsis prediction and proteomic data interpretation^13^. Despite strengths—including detecting non-linear relationships in high-dimensional data, scalable deployment in resource-limited settings, and automated preprocessing of microbiological workflows—key limitations persisted: opaque “black-box” models eroded clinical trust, few tools addressed limb-threatening infections like necrotizing fasciitis, and insufficient real-world validation hindered clinical utility. Explainable AI (XAI) frameworks emerged to clarify decision-making processes, bridging algorithmic performance and practical integration into diagnostics.

Severe limb infections like NF and OM require rapid, accurate differentiation, but overlapping symptoms and diagnostic limitations (delays, costs, invasiveness) hinder early intervention, worsening outcomes. Routine blood biomarkers offer accessible, timely data reflecting systemic responses. We explored ML using these biomarkers for rapid differentiation. However, “black-box” models lack transparency. Therefore, we developed and validated an XAI model using only routine blood tests to distinguish NF from OM. This approach aims to provide trustworthy, rapid risk stratification, elucidate key differentiating biomarkers, build clinician confidence, and ultimately improve patient management in these critical infections.

## Results

### Study population

**Fig. 1:**
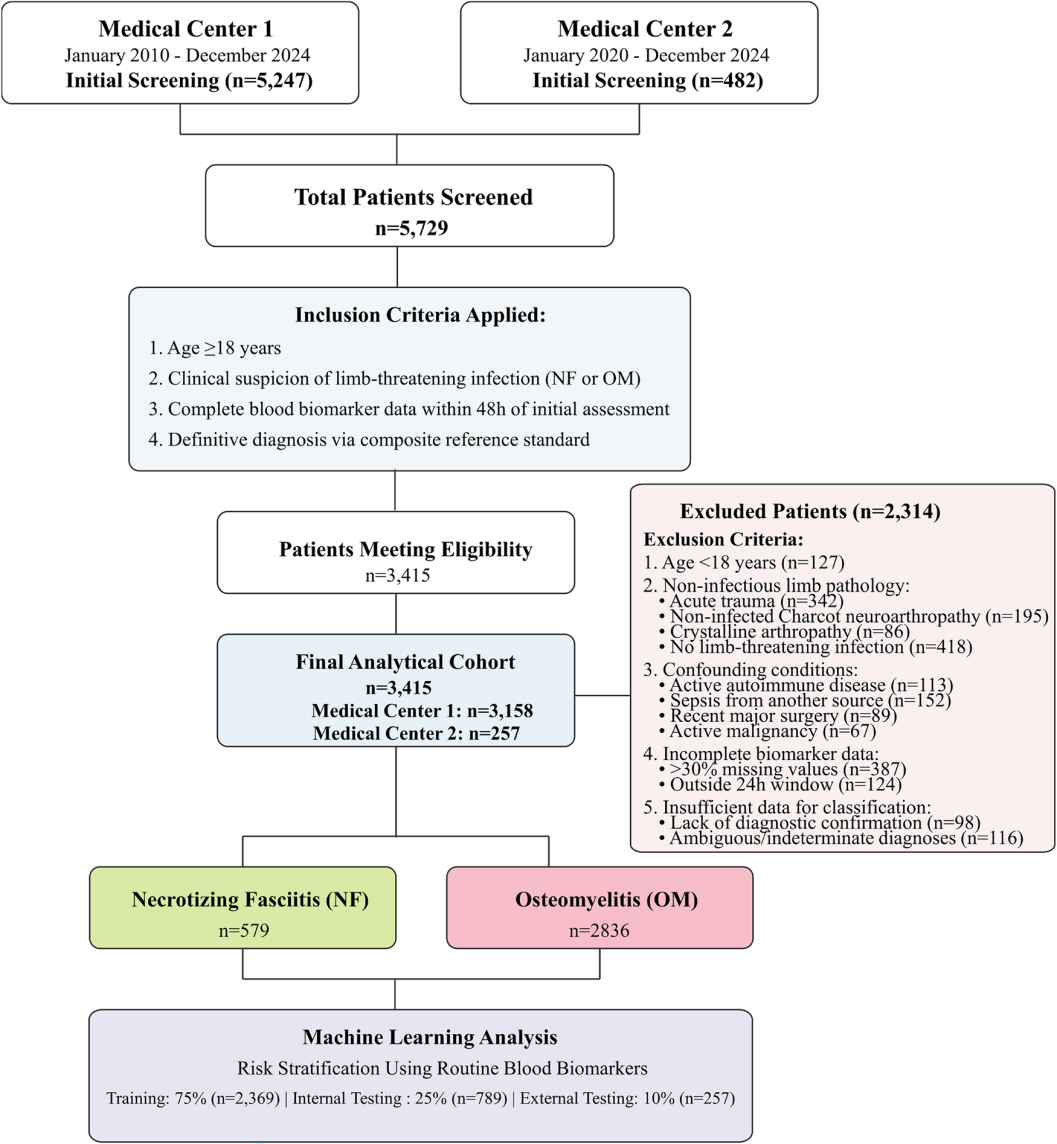
Enrollment flowchart illustrating patient selection from two clinical centers. Demonstrates the patient screening, eligibility assessment, and exclusion process across the two participating clinical centers.

As detailed in the study flowchart (Fig. 1), this retrospective analysis included a total of 3415 patients from two medical centers within the same geographic region (Xinjiang Uyghur Autonomous Region). The development cohort was sourced from Center 1 (The Sixth Affiliated Hospital of Xinjiang Medical University), comprising 3158 patients, while the external validation cohort consisted of 257 patients from Center 2 (The First People’s Hospital of Kashi Prefecture). Across both centers, the final study population included 579 individuals with a final diagnosis of NF and 2836 with osteomyelitis. The baseline characteristics of these cohorts revealed significant demographic and clinical differences (Table 1). Patients with NF were, on average, significantly older than those with osteomyelitis (mean age 54.6 vs. 42.7 years, *P* < 0.001) and were more likely to be male (73.2% vs. 67.4%, *P* = 0.007). The NF cohort presented a markedly more severe systemic inflammatory response, evidenced by significantly higher white blood cell and neutrophil counts, C-reactive protein (CRP), and erythrocyte sedimentation rate (ESR), alongside lower lymphocyte counts (all *P* < 0.001). This acute systemic insult was further reflected in profound metabolic derangements, most notably severe hypoalbuminemia (mean 31.2 vs. 37.9 g/L) and hypocalcemia (mean 1.87 vs. 2.07 mmol/L), as well as hyponatremia and dyslipidemia characterized by lower total cholesterol, HDL, and LDL (all *P* < 0.001). Furthermore, patients with NF exhibited greater evidence of end-organ stress, including elevated creatinine, total bilirubin, and liver transaminases, coupled with a more pronounced anemia compared to the osteomyelitis group (all *P* < 0.05). Collectively, these baseline data delineated a distinct pathophysiological signature for necrotizing fasciitis, characterized by a fulminant systemic inflammatory cascade and multisystem metabolic dysfunction that starkly contrasted with the profile observed in patients with osteomyelitis. To assess the comparability of the two cohorts, we performed a comprehensive baseline characteristics comparison stratified by center. Supplementary Table S1 presents demographic and clinical characteristics of patients from Center 1 (*N* = 3158) and Center 2 (*N* = 257). The two cohorts demonstrate highly similar patient demographics and clinical feature distributions across all variables, with no statistically significant differences in most parameters. Only AST (*P* = 0.003) and ALT (*P* < 0.001) showed statistically significant but small differences between centers, supporting the appropriateness of external validation for assessing model generalizability.

**Table 1:** Patients baseline characteristics

Features	ALL *N* = 3415	NF *N* = 579	Osteo *N* = 2836	*P*
Age	44.7 ± 18.3	54.6 ± 14.9	42.7 ± 18.3	<0.001
Gender:				0.007
Female	1079 (31.6%)	155 (26.8%)	924 (32.6%)	
Male	2336 (68.4%)	424 (73.2%)	1912 (67.4%)	
WBC	9.07 ± 5.25	13.0 ± 8.24	8.26 ± 3.93	<0.001
Lymphocyt	1.99 ± 1.48	1.55 ± 2.58	2.08 ± 1.12	<0.001
Neutrophil	6.20 ± 4.85	10.4 ± 7.41	5.34 ± 3.57	<0.001
Monocyte	0.68 ± 0.86	0.92 ± 1.92	0.62 ± 0.34	<0.001
Basophils	0.03 ± 0.02	0.04 ± 0.03	0.03 ± 0.02	<0.001
Eosinophils	0.15 ± 0.15	0.10 ± 0.12	0.16 ± 0.15	<0.001
HB	120 ± 23.8	109 ± 25.6	123 ± 22.8	<0.001
Hematocrit	35.8 ± 9.48	33.4 ± 7.46	36.3 ± 9.77	<0.001
Platelet	315 ± 129	302 ± 137	317 ± 127	0.012
ESR	35.6 ± 14.4	37.2 ± 8.19	35.3 ± 15.4	<0.001
CRP	36.5 ± 36.0	45.5 ± 38.6	34.7 ± 35.2	<0.001
CK	153 ± 594	211 ± 869	141 ± 520	0.061
CK_MB	16.8 ± 8.43	16.4 ± 2.27	16.9 ± 9.19	0.012
TC	3.74 ± 0.81	3.53 ± 0.82	3.78 ± 0.80	<0.001
TG	1.39 ± 0.86	1.53 ± 0.83	1.37 ± 0.87	<0.001
K	3.85 ± 0.54	3.74 ± 0.56	3.87 ± 0.54	<0.001
Na	138 ± 4.73	136 ± 5.41	139 ± 4.39	<0.001
Ca	2.04 ± 0.47	1.87 ± 0.48	2.07 ± 0.46	<0.001
Cl	104 ± 4.85	102 ± 5.90	104 ± 4.55	<0.001
Mg	0.85 ± 0.10	0.86 ± 0.13	0.85 ± 0.09	0.086
P	1.27 ± 0.32	1.14 ± 0.37	1.30 ± 0.30	<0.001
Creatinine	72.7 ± 126	92.8 ± 124	68.7 ± 126	<0.001
eGFR	110 ± 17.9	109 ± 13.7	110 ± 18.7	0.023
Uacid	281 ± 168	279 ± 211	281 ± 157	0.845
CYS_C	0.92 ± 0.36	0.98 ± 0.50	0.91 ± 0.32	0.001
HbA1c	7.98 ± 1.39	8.51 ± 1.54	7.87 ± 1.33	<0.001
TP	69.5 ± 8.47	65.6 ± 10.2	70.3 ± 7.82	<0.001
ALB	36.8 ± 6.43	31.2 ± 6.97	37.9 ± 5.68	<0.001
AST	32.1 ± 60.5	49.6 ± 129	28.5 ± 30.7	<0.001
ALT	29.1 ± 50.3	38.6 ± 78.3	27.2 ± 42.0	0.001
AFU	20.0 ± 5.27	19.6 ± 2.34	20.0 ± 5.68	0.004
NT_5	5.98 ± 3.64	6.46 ± 5.45	5.88 ± 3.14	0.013
HDL	0.93 ± 0.29	0.78 ± 0.27	0.97 ± 0.29	<0.001
LDL	2.42 ± 0.64	2.26 ± 0.62	2.45 ± 0.64	<0.001
GGT	41.8 ± 49.1	61.3 ± 65.4	37.8 ± 44.1	<0.001
ALP	119 ± 71.5	129 ± 87.3	117 ± 67.7	0.001
LDH	251 ± 679	308 ± 1146	239 ± 535	0.159
DBIL	2.25 ± 7.44	2.96 ± 13.2	2.10 ± 5.57	0.124
IBIL	6.95 ± 5.99	7.91 ± 8.19	6.75 ± 5.42	0.001
TBIL	12.8 ± 14.1	18.7 ± 24.1	11.5 ± 10.6	<0.001
UMAlb	353 ± 84.9	354 ± 90.0	353 ± 83.8	0.756
UACR	628 ± 62.7	627 ± 75.2	628 ± 59.9	0.717

In Table 1, all continuous variables are presented as mean ± standard deviation (SD), implying an assumption of approximate normality, while categorical variables are expressed as counts with percentages (*n* (%)). The full names and units of the variables are as follows: Age (years); Gender (Female/Male); WBC – white blood cell count (×10⁹/L); Lymphocyt – lymphocyte count (×10⁹/L); Neutrophil – neutrophil count (×10⁹/L); Monocyte – monocyte count (×10⁹/L); Basophils – basophil count (×10⁹/L); Eosinophils – eosinophil count (×10⁹/L); HB – hemoglobin concentration (g/L); Hematocrit – volume percentage of red blood cells in blood (%); Platelet – platelet count (×10⁹/L); ESR – erythrocyte sedimentation rate (mm/h); CRP – C-reactive protein (mg/L); CK – creatine kinase (U/L); CK_MB – creatine kinase-MB isoenzyme (U/L); TC – total cholesterol (mmol/L); TG – triglycerides (mmol/L); K – potassium (mmol/L); Na – sodium (mmol/L); Ca – calcium (mmol/L); Cl – chloride (mmol/L); Mg – magnesium (mmol/L); P – phosphorus/inorganic phosphate (mmol/L); Creatinine – serum creatinine (μmol/L); eGFR – estimated glomerular filtration rate (mL/min/1.73 m²); Uacid – uric acid (μmol/L); CYS_C – cystatin C (mg/L); HbA1c – glycated hemoglobin A1c (%); TP – total protein (g/L); ALB – albumin (g/L); AST – aspartate aminotransferase (U/L); ALT – alanine aminotransferase (U/L); AFU – alpha-L-fucosidase (U/L); NT_5 – 5’-nucleotidase (U/L); HDL – high-density lipoprotein cholesterol (mmol/L); LDL – low-density lipoprotein cholesterol (mmol/L); GGT – gamma-glutamyl transferase (U/L); ALP – alkaline phosphatase (U/L); LDH – lactate dehydrogenase (U/L); DBIL – direct (conjugated) bilirubin (μmol/L); IBIL – indirect (unconjugated) bilirubin (μmol/L); TBIL – total bilirubin (μmol/L); UMAlb – urinary microalbumin (mg/L); and UACR – urinary albumin-to-creatinine ratio (mg/g or mg/mmol). The white blood cell subtypes (lymphocytes, neutrophils, monocytes, eosinophils, basophils) are standard components of a complete blood count (CBC) differential.

### Systematic feature selection and model fitting

**Fig. 2:**
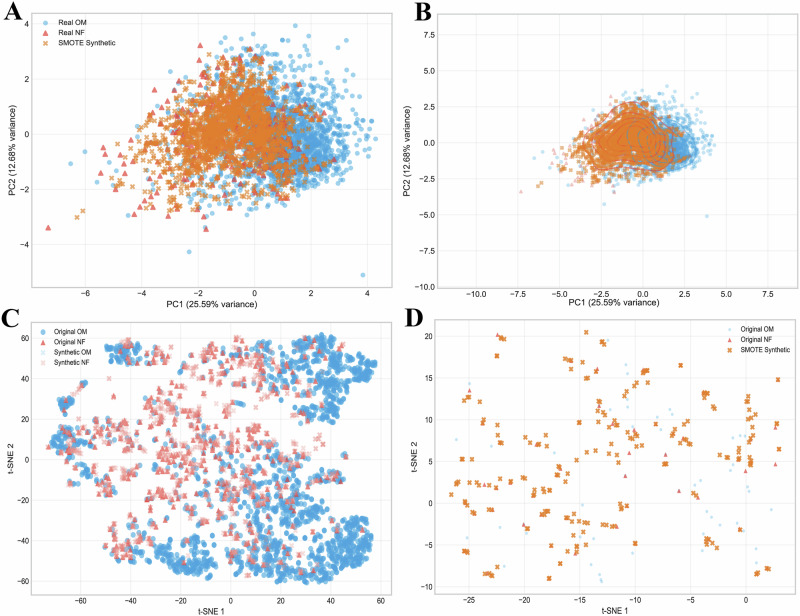
PCA and t-SNE visualization of SMOTE synthetic samples. **A** Scatter plot of the first two principal components (PC1 and PC2) showing the distribution of real Osteomyelitis (OM), real Necrotizing Fasciitis (NF), and synthetic NF samples; **B** Kernel density estimation (KDE) contours illustrating the probabilistic overlap and feature space density of the three sample groups; **C** t-SNE projection of the full training dataset using a perplexity of 30, visualizing high-dimensional relationships in a two-dimensional embedding; **D** High-magnification t-SNE view of the minority class cluster focusing on the local neighborhood consistency between real and synthetic observations.

Prior to the main modeling pipeline, a comprehensive comparison was performed to select the most effective class balancing strategy. As illustrated in Supplementary Fig. 1, eight different resampling techniques were evaluated across all base classifiers in a cross-validation framework. The results consistently demonstrated that the SMOTE provided superior performance across all key metrics. Specifically, SMOTE achieved the highest mean AUC of 0.843 and the highest mean F1-score of 0.760. Based on this empirical evidence, SMOTE was confirmed as the definitive resampling method for the training cohort in all subsequent stages of the study. To provide empirical support for the clinical plausibility of SMOTE-generated synthetic samples and to verify that our chosen resampling strategy outperforms alternatives, we conducted a series of rigorous sensitivity analyses grounded in low-dimensional visualizations. Principal Component Analysis (PCA) of the dataset across 44 clinical and laboratory features revealed that the first two principal components captured 38.27% of the total variance, with real majority class samples (other soft tissue infections, OM; blue circles) and real minority class samples (necrotizing fasciitis, NF; red triangles) occupying distinct yet partially overlapping regions in feature space (Fig. 2A). Critically, synthetic NF samples generated via SMOTE (orange triangles) were not only positioned within the manifold of real NF cases but also strategically filled sparse regions of the minority class distribution, extending toward the decision boundary with the OM class as intended. Kernel density estimation further corroborated this spatial alignment, showing that the contours of synthetic samples closely mirrored the biological patterns of authentic NF cases while effectively bridging gaps between disparate real instances (Fig. 2B). Complementary t-distributed Stochastic Neighbor Embedding (t-SNE) analysis preserved local neighborhood structures and reinforced these findings: synthetic NF samples integrated seamlessly into the existing NF manifold without forming isolated or anomalous clusters (Fig. 2C). A magnified view of the NF-dominant region confirmed that synthetic instances enhanced minority class density without spilling into or distorting the OM feature space (Fig. 2D). Collectively, these visual and statistical validations demonstrate that SMOTE-generated samples maintain the intrinsic biological and clinical architecture of real necrotizing fasciitis cases, thereby supporting their clinical plausibility and justifying their incorporation into downstream classification models aimed at early, accurate diagnosis in imbalanced clinical settings.

**Fig. 3:**
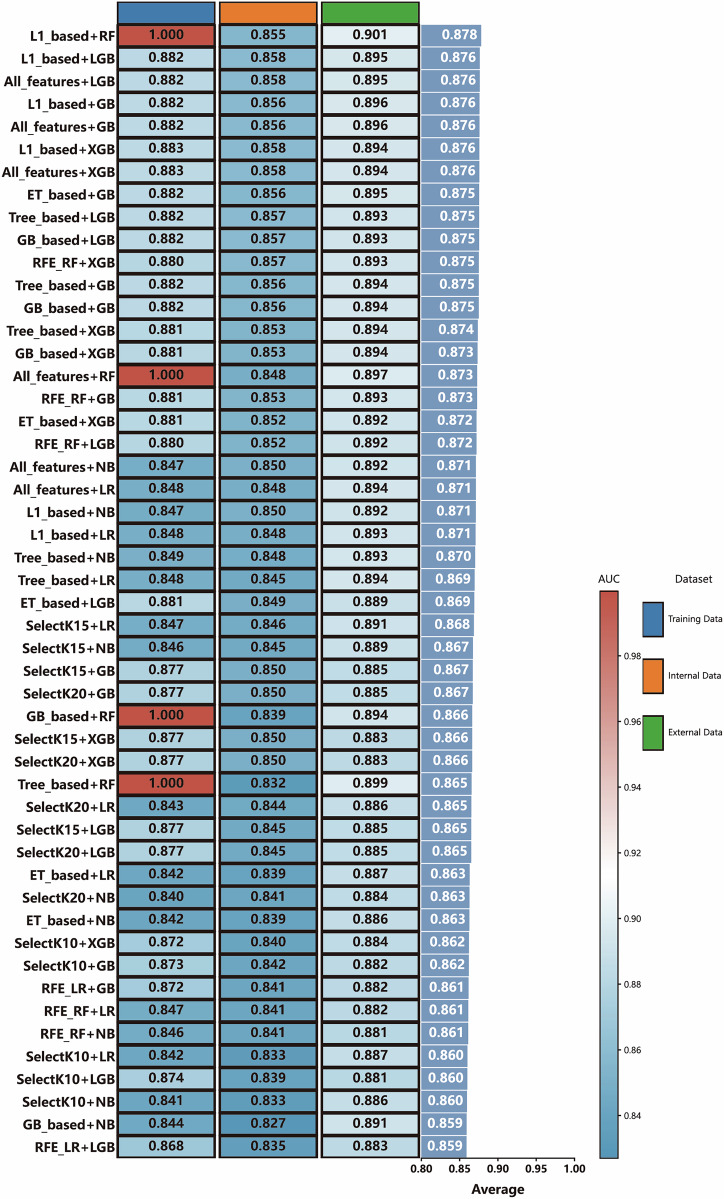
Performance comparison of top 50 machine learning algorithm combinations across training, internal validation, and external validation datasets. The heatmap visualizes the comparative performance of different machine learning models based on the Area Under the Curve (AUC) metric; the rows represent unique combinations of a feature selection method (e.g., L1-based, All features, Tree-based) and a classification algorithm (e.g., RF, LGB, GB, XGB), while the columns correspond to the performance on the training data, internal validation data, and external validation data, respectively; the numerical value within each cell indicates the specific AUC achieved, and the cell’s color provides a visual representation of this value, with warmer colors denoting higher AUCs as defined by the color bar legend on the right; the final column presents the average AUC for each model across the three datasets, serving as an overall performance summary.

Our comprehensive evaluation of various feature selection and machine learning algorithm combinations revealed that the L1-based feature selection method paired with a RF classifier achieved the highest average performance across all datasets (Fig. 3). This L1-based+RF model yielded an average AUC of 0.878, although it demonstrated perfect classification on the training data with an AUC of 1.000, its performance generalized well with an AUC of 0.855 on the internal validation set and a robust 0.901 on the external validation set. Several other models also showed strong, comparable performance, each achieving an average AUC of 0.876. These included the LGBM and GB models when using either L1-based feature selection or all available features. For instance, the L1-based+LGB model recorded AUCs of 0.882, 0.858, and 0.895 on the training, internal, and external datasets, respectively. Similarly, the combination of all features with an XGB classifier produced a high average AUC of 0.876, with specific AUCs of 0.883 on the training set, 0.858 on the internal validation set, and 0.894 on the external validation set. The top-performing models consistently utilized ensemble methods like RF, LGBM, GB, and XGB, underscoring their effectiveness for this clinical differentiation task.

**Fig. 4:**
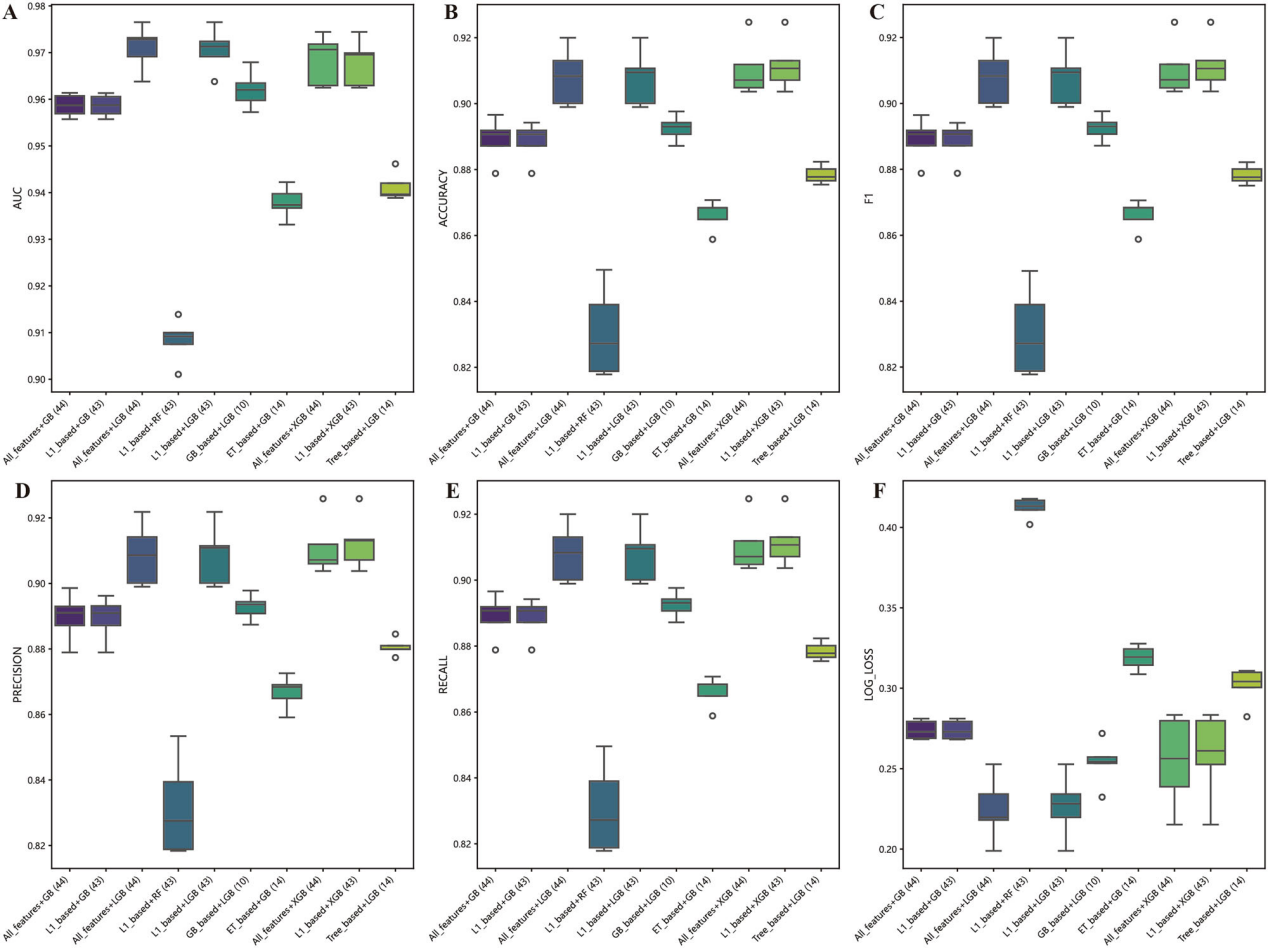
Distribution of performance metrics during nested cross-validation tuning. Six box plots displaying key performance metrics (AUC, accuracy, F1, precision, recall, and log loss) for the top 10 machine learning models across validation folds; Each box plot shows median performance (horizontal line), interquartile range (box), and outliers (circles) for different feature selection and algorithm combinations. **A** Boxplot illustrating the distribution of Area Under the Curve (AUC) values for each model-feature selector combination across the inner cross-validation folds; **B** Distribution of accuracy scores achieved by each model configuration; **C** Distribution of F1 scores, representing the harmonic mean of precision and recall; **D** Distribution of precision values; **E** Distribution of recall values; **F** Distribution of log loss, a metric for prediction error. Each plot’s x-axis identifies the model architecture and the feature selection method used, with the number of features in parentheses, while the y-axis represents the scale for the respective performance metric, and the boxplots visualize the median, interquartile range, and outliers for each configuration during the tuning process.

Our comprehensive nested cross-validation process revealed distinct performance patterns across the top 10 machine learning models evaluated for differentiating necrotizing fasciitis from osteomyelitis. As shown in Fig. 4 and Table 2, gradient boosting-based approaches consistently demonstrated superior performance across multiple evaluation metrics. The LGB with all features achieved the highest cross-validation AUC (0.971 ± 0.005), accuracy (0.908 ± 0.009), and F1 score (0.908 ± 0.009), while maintaining excellent precision (0.909 ± 0.010) and recall (0.908 ± 0.009). XGBoost models with either L1-based feature selection (43 features) or all features (44 features) delivered comparable performance, with AUCs of 0.968 ± 0.005 and 0.969 ± 0.005, respectively. Notably, models using L1-based feature selection maintained performance comparable to those using all features, suggesting effective dimensionality reduction without significant information loss. The model with the most parsimonious feature set, LGB with GB-based selection (10 features), still achieved an impressive AUC of 0.962 ± 0.004, demonstrating robust performance with significantly reduced complexity.

**Table 2:** Detailed information of nested cross validation tuning process

Model name	Feature selector	Feature count	AUC	Accuracy	F1	Precision	Recall	Log loss	Brier loss
GB	All features	44	0.9587 ± 0.0024	0.8890 ± 0.0066	0.8890 ± 0.0066	0.8897 ± 0.0073	0.8890 ± 0.0066	0.2741 ± 0.0059	0.0821 ± 0.0026
GB	L1_based	43	0.9586 ± 0.0024	0.8886 ± 0.0060	0.8885 ± 0.0060	0.8893 ± 0.0067	0.8886 ± 0.0060	0.2740 ± 0.0060	0.0822 ± 0.0025
LGB	All features	44	0.9711 ± 0.0048	0.9081 ± 0.0089	0.9081 ± 0.0088	0.9087 ± 0.0096	0.9081 ± 0.0089	0.2247 ± 0.0201	0.0679 ± 0.0064
RF	L1 based	43	0.9083 ± 0.0047	0.8305 ± 0.0136	0.8304 ± 0.0135	0.8315 ± 0.0149	0.8305 ± 0.0136	0.4121 ± 0.0064	0.1275 ± 0.0030
LGB	L1 based	43	0.9706 ± 0.0047	0.9079 ± 0.0086	0.9078 ± 0.0086	0.9086 ± 0.0094	0.9079 ± 0.0086	0.2268 ± 0.0197	0.0685 ± 0.0063
LGB	GB based	10	0.9621 ± 0.0040	0.8926 ± 0.0039	0.8926 ± 0.0039	0.8928 ± 0.0039	0.8926 ± 0.0039	0.2538 ± 0.0142	0.0773 ± 0.0035
GB	ET based	14	0.9378 ± 0.0034	0.8662 ± 0.0046	0.8662 ± 0.0046	0.8668 ± 0.0051	0.8662 ± 0.0046	0.3189 ± 0.0076	0.0985 ± 0.0032
XGB	All features	44	0.9685 ± 0.0054	0.9104 ± 0.0086	0.9104 ± 0.0086	0.9109 ± 0.0088	0.9104 ± 0.0086	0.2547 ± 0.0286	0.0716 ± 0.0072
XGB	L1 based	43	0.9679 ± 0.0051	0.9119 ± 0.0080	0.9118 ± 0.0080	0.9126 ± 0.0084	0.9119 ± 0.0080	0.2584 ± 0.0273	0.0720 ± 0.0073
LGB	Tree based	14	0.9412 ± 0.0030	0.8785 ± 0.0028	0.8783 ± 0.0028	0.8805 ± 0.0026	0.8785 ± 0.0028	0.3015 ± 0.0116	0.0906 ± 0.0034

Precision, recall, and F1-score were computed using macro averaging (average = ‘macro’ in scikit-learn), which assigns equal weight to both classes—necrotizing fasciitis and osteomyelitis—to ensure balanced performance evaluation despite class imbalance.

*AUC* AUC stands for Area Under the Receiver Operating Characteristic (ROC) Curve, *95% CI* 95% confidence intervals of AUC, *GB* Gradient Boosting, *LGB* LightGBM, *RF* Random Forest, *XGB* XGBoost.

### Performance of each tuned model combination

**Fig. 5:**
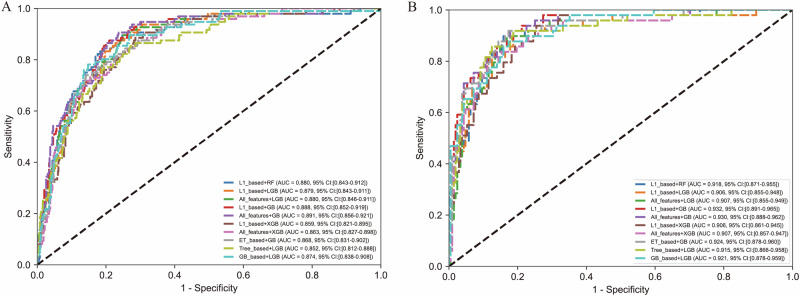
Discriminative performance of tuned models on internal and external test sets. **A** Receiver Operating Characteristic (ROC) curves for the top 10 performing models evaluated on the internal testing dataset, where each curve plots the true positive rate (sensitivity) against the false positive rate (1-specificity) at various threshold settings; **B** ROC curves for the same ten models evaluated on the independent external testing dataset, with the legend for both panels detailing each model-feature selector combination and its corresponding Area Under the Curve (AUC) with 95% confidence intervals.

The discriminative performance of the ten optimized models was rigorously assessed on both an internal and an independent external testing cohort, with results visualized as ROC curves (Fig. 5) and detailed in Tables 3 and 4. On the internal testing set, all models demonstrated strong predictive capability, with AUC values clustered between 0.852 and 0.891 (Fig. 5A). The GB model using all 44 features achieved the highest AUC of 0.891, complemented by an accuracy of 0.844 and F1-score of 0.747 (Table 3). When subjected to the more challenging external testing cohort validation, the models not only maintained but significantly improved their performance, underscoring their robustness and generalizability (Fig. 5B). The top-performing model on this external dataset was the GB classifier with 43 features selected via L1 regularization, which attained an exceptional AUC of 0.932 (95% CI: 0.891–0.965), an accuracy of 0.879, and a Brier loss of 0.084, indicating superior discrimination and calibration (Table 4). Critically, the parsimonious LGB model, developed using only 10 features identified by the GB-based selector, also delivered outstanding results with an AUC of 0.921 (95% CI: 0.878–0.959), a balanced F1-score of 0.800, and the lowest Brier loss among all models at 0.081. While the GB model using 43 features achieved the highest AUC (0.932), we identified the LGB model using the 10-feature subset as the optimal model for further analysis and potential clinical deployment. Our rationale is based on a holistic evaluation that prioritizes not just predictive accuracy but also clinical parsimony and reliability. This 10-feature model demonstrated outstanding and highly comparable performance with an AUC of 0.921, but more importantly, it achieved the lowest Brier loss (0.081) and a low Log loss (0.264), indicating superior calibration and more trustworthy probability estimates. The principle of parsimony is critical for real-world application; a 10-feature model is far more practical and likely to be adopted in a clinical setting than a more cumbersome 43-feature model. Therefore, this balanced and high-performing 10-feature LGB model was selected for the subsequent explainability analysis. The selected features were Age, Gender, Neutrophil, Basophils, ESR, CRP, Ca, ALB, HDL, and TBIL.

**Table 3:** Tuned performance of models on internal testing dataset

Model name	Feature selector	Feature count	AUC	95% CI	Accuracy	F1	Precision	Recall	Log loss	Brier loss
GB	All features	44	0.891	0.856–0.921	0.844	0.747	0.725	0.785	0.323	0.102
GB	L1 based	43	0.888	0.852–0.919	0.836	0.734	0.713	0.772	0.330	0.104
LGB	All features	44	0.880	0.846–0.911	0.849	0.736	0.725	0.750	0.338	0.107
RF	L1 based	43	0.880	0.843–0.912	0.822	0.736	0.710	0.806	0.421	0.132
LGB	L1 based	43	0.879	0.843–0.911	0.858	0.748	0.738	0.759	0.341	0.107
LGB	GB based	10	0.874	0.838–0.908	0.849	0.732	0.723	0.742	0.331	0.103
GB	ET based	14	0.868	0.831–0.902	0.839	0.747	0.722	0.795	0.370	0.119
XGB	All features	44	0.863	0.827–0.898	0.849	0.725	0.722	0.729	0.414	0.115
XGB	L1 based	43	0.859	0.821–0.895	0.846	0.730	0.719	0.744	0.425	0.122
LGB	Tree based	14	0.852	0.812–0.888	0.822	0.720	0.698	0.764	0.412	0.128

Precision, recall, and F1-score were computed using macro averaging (average = ‘macro’ in scikit-learn), which assigns equal weight to both classes—necrotizing fasciitis and osteomyelitis—to ensure balanced performance evaluation despite class imbalance.

*AUC* AUC stands for Area Under the Receiver Operating Characteristic (ROC) Curve, *95% CI* 95% confidence intervals of AUC, *GB* Gradient Boosting, *LGB* LightGBM, *RF* Random Forest, *XGB* XGBoost.

**Table 4:** Tuned performance of models on external testing dataset

Model name	Feature selector	Feature count	AUC	95% CI	Accuracy	F1	Precision	Recall	Log loss	Brier loss
GB	All features	44	0.930	0.888–0.962	0.883	0.819	0.807	0.834	0.281	0.084
GB	L1 based	43	0.932	0.891–0.965	0.879	0.817	0.800	0.840	0.280	0.084
LGB	All features	44	0.907	0.855–0.949	0.887	0.803	0.831	0.782	0.299	0.089
RF	L1 based	43	0.918	0.871–0.955	0.852	0.798	0.770	0.862	0.392	0.120
LGB	L1 based	43	0.906	0.855–0.948	0.883	0.801	0.818	0.787	0.297	0.090
LGB	GB based	10	0.921	0.878–0.959	0.879	0.800	0.808	0.793	0.264	0.081
GB	ET based	14	0.924	0.878–0.960	0.864	0.804	0.779	0.846	0.303	0.092
XGB	All features	44	0.907	0.857–0.947	0.875	0.772	0.820	0.744	0.340	0.092
XGB	L1 based	43	0.906	0.861–0.945	0.864	0.770	0.783	0.760	0.349	0.100
LGB	Tree based	14	0.915	0.866–0.958	0.879	0.814	0.801	0.832	0.291	0.087

Precision, recall, and F1-score were computed using macro averaging (average = ‘macro’ in scikit-learn), which assigns equal weight to both classes—necrotizing fasciitis and osteomyelitis—to ensure balanced performance evaluation despite class imbalance.

*AUC* AUC stands for Area Under the Receiver Operating Characteristic (ROC) Curve, *95% CI* 95% confidence intervals of AUC, *GB* Gradient Boosting, *LGB* LightGBM, *RF* Random Forest, *XGB* XGBoost.

**Fig. 6:**
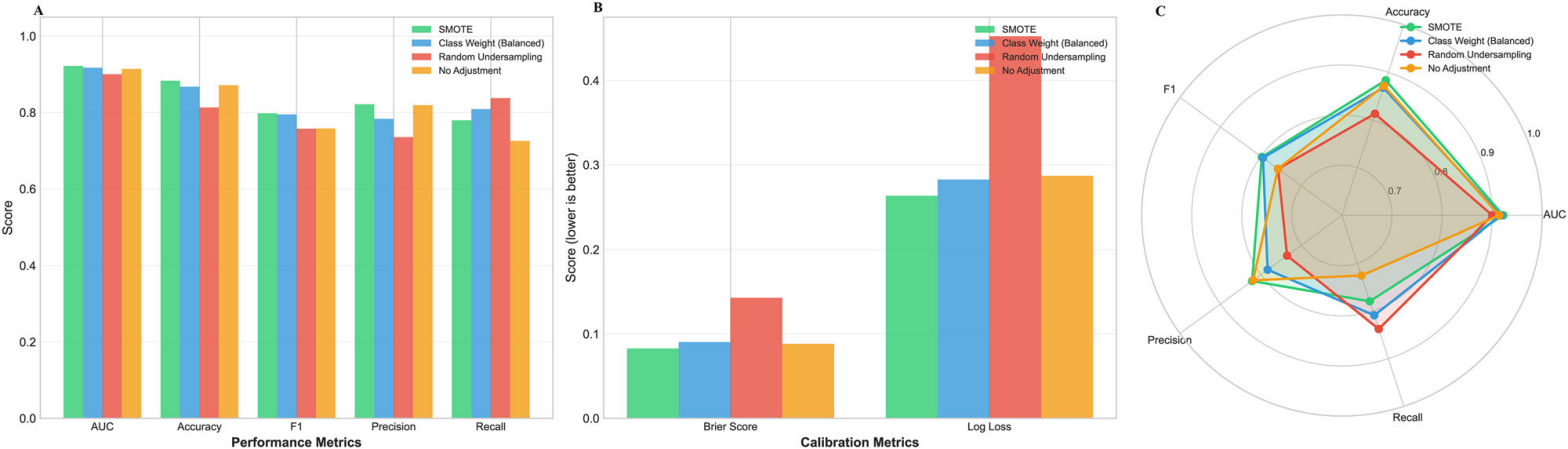
Comparative assessment of class imbalance handling strategies for the diagnostic model. **A** Bar charts illustrating comparative discrimination performance across five key statistical metrics for four imbalance adjustment techniques; **B** Graphical representation of calibration metrics where lower values signify enhanced alignment between predicted probabilities and observed clinical outcomes; **C** Radar chart providing a multidimensional visualization of strategy performance, mapping the relative strengths of each approach across various diagnostic parameters to identify the most balanced classification method.

To optimize the predictive accuracy of our diagnostic model for distinguishing necrotizing fasciitis from osteomyelitis, we conducted a rigorous comparative evaluation of four class imbalance handling strategies—SMOTE, balanced class weighting, random undersampling, and no adjustment—within a 10-feature LightGBM framework on an external validation cohort (*n* = 257). Direct comparison confirmed that SMOTE delivered superior balanced performance across both discrimination and calibration metrics (Fig. 6). On the external validation set, SMOTE achieved the highest AUC and F1-score, outperforming all alternative approaches. Further assessment of discrimination revealed that SMOTE consistently attained the best results across AUC, accuracy, F1-score, precision, and recall, demonstrating an optimal balance between sensitivity and specificity in a clinical setting (Fig. 6A). In terms of calibration, SMOTE yielded the lowest Brier score and a highly competitive log loss, indicating that its predicted probabilities more accurately reflected true clinical risk (Fig. 6B). A radar chart integrating these performance indicators visually reinforced these findings, showing that the SMOTE-based model occupied the largest functional area, with particularly pronounced advantages in F1-score and precision relative to traditional undersampling or weighting methods (Fig. 6C). Collectively, these results establish SMOTE as the most robust strategy for this imbalanced classification task, ensuring both high diagnostic discrimination and reliable probability estimation to support clinical decision-making.

**Fig. 7:**
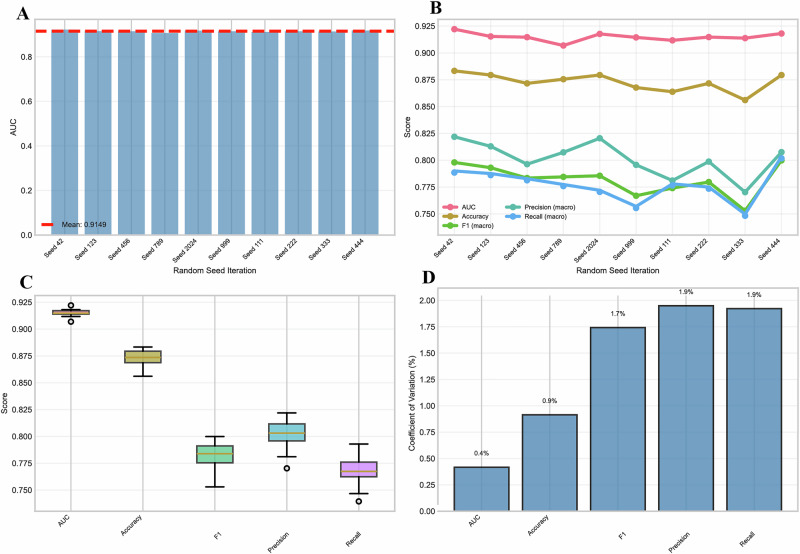
Stability analysis of the predictive model across random initializations. **A** Individual and mean performance values for the primary discrimination index across multiple stochastic iterations; **B** Longitudinal tracking of multiple performance metrics illustrating the consistency of model behavior across different computational seeds; **C** Distributional analysis showing the variance and median values for each diagnostic metric to assess the range of potential performance fluctuations; **D** Comparative analysis of the coefficient of variation for each metric, serving as a standardized measure of model robustness where lower percentages represent higher architectural stability.

Evaluation of model stability across 10 different random seeds demonstrated exceptional robustness to stochastic variation. Using the external validation cohort, we assessed the model under ten distinct random seed initializations (Fig. 7). The Area Under the Curve (AUC) remained remarkably consistent across all iterations, with values tightly clustered around a mean of 0.9149 and minimal fluctuation between individual seeds (Fig. 7A). This consistency extended to all primary discrimination metrics—Accuracy, F1-score, Precision, and Recall—which exhibited nearly overlapping trajectories across seed iterations (Fig. 7B). Box plot distributions further corroborated this stability, revealing highly concentrated interquartile ranges and an especially narrow spread for AUC, indicating that the model’s predictive performance was independent of specific stochastic initializations (Fig. 7C). Quantitative assessment confirmed these observations: the coefficient of variation (CV%) for AUC was only 0.4%, and all other metrics remained well below the 2.0% threshold (Fig. 7D). Collectively, these results established that the model’s diagnostic performance was highly reproducible and reflected genuine biological signals in the blood biomarker data rather than computational artifacts.

**Fig. 8:**
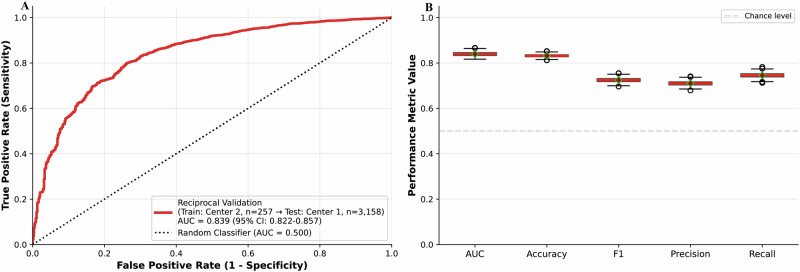
Reciprocal validation of the diagnostic model across dual-center datasets. **A** Receiver Operating Characteristic (ROC) curve for the model trained on Center 2 data and tested on Center 1 data, illustrating the trade-off between sensitivity and specificity in a larger independent cohort; **B** Bootstrap distributions of five core performance metrics on the reciprocal validation set, providing a statistical summary of model reliability and the variance of key diagnostic indicators.

We performed a reciprocal validation experiment to assess the generalizability of our diagnostic framework by training the same model on the smaller Center 2 cohort (*n* = 257) and evaluating its performance on the larger, independent Center 1 dataset (*n* = 3158). In this setting, the model achieved an area under the receiver operating characteristic curve (AUC) of 0.840 (95% CI: 0.822–0.857), with an accuracy of 0.832, F1-score of 0.725, precision of 0.710, and recall of 0.746—significantly outperforming a random classifier (Fig. 8A). For direct comparison, the original model trained on Center 1 and tested on Center 2 yielded an AUC of 0.921 (95% CI: 0.878–0.959), accuracy of 0.879, F1-score of 0.800, precision of 0.808, and recall of 0.793. Bootstrap analysis of the external validation further confirmed the model’s stability, with distributions of AUC, accuracy, F1-score, precision, and recall consistently concentrated well above chance level (Fig. 8B).

### Threshold optimalization and assessment

**Fig. 9:**
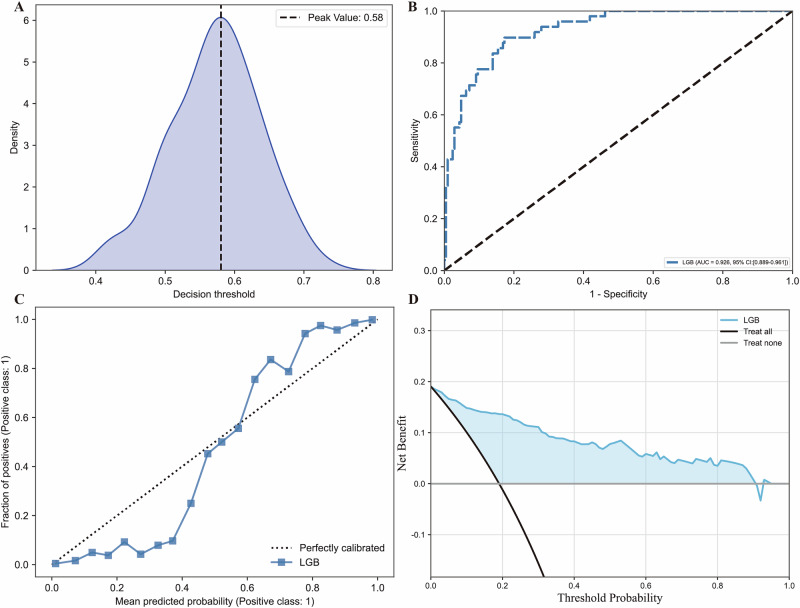
Performance and clinical utility of the optimized LGB model on the external test set. **A** A kernel density plot showing the distribution of optimal decision thresholds determined during model tuning, with the peak value indicated by a dashed vertical line; **B** The Receiver Operating Characteristic (ROC) curve of the final model, plotting sensitivity against 1-specificity and providing the Area Under the Curve (AUC) with its 95% confidence interval; **C** A calibration plot comparing the mean predicted probabilities (x-axis) with the observed fraction of positive cases (y-axis), benchmarked against a perfectly calibrated diagonal line; **D** A decision curve analysis plotting the net benefit of using the model’s predictions across a spectrum of threshold probabilities against the strategies of treating all patients or treating none.

To optimize clinical utility, the decision threshold of the final 10-feature LightGBM model was tuned on the combined training and internal validation sets, identifying an optimal cutoff of 0.58 based on Youden’s J statistic (Fig. 9A). After refitting the model with this new threshold, its performance was definitively assessed on the independent external validation cohort. The model demonstrated excellent discriminative ability, achieving an AUC of 0.926 (95% CI: 0.889–0.961), significantly outperforming random chance (Fig. 9B). The calibration plot revealed that while the model’s predictions generally followed the ideal diagonal, it tended to slightly underestimate the risk for probabilities above 0.6 and slightly overestimate risk in the 0.1–0.4 range, indicating reasonable but imperfect calibration (Fig. 9C). Critically, the decision curve analysis confirmed the model’s clinical value, showing a consistent positive net benefit across a wide and clinically relevant range of threshold probabilities from ~0.15 to 0.90 (Fig. 9D). This result indicated that using the model to inform clinical decisions would be superior to the default strategies of treating all patients or treating none for necrotizing fasciitis.

**Fig. 10:**
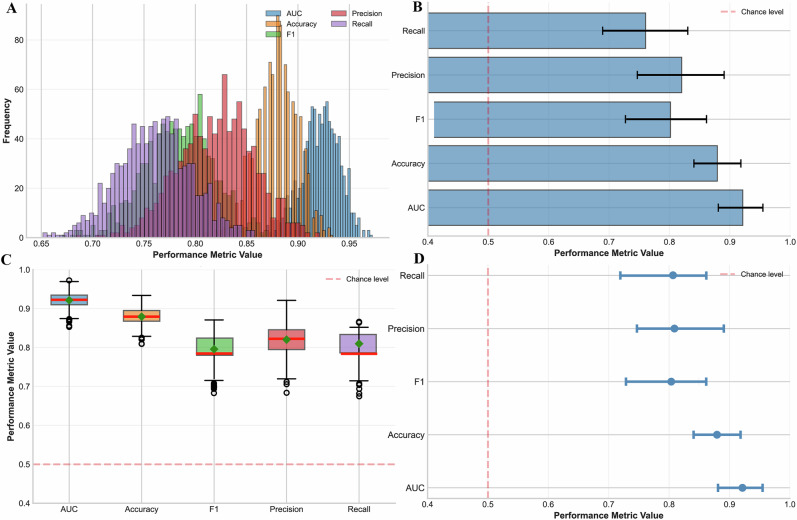
Comprehensive bootstrap resampling analysis for external validation. **A** Histograms representing the frequency distribution of performance metrics derived from 1000 bootstrap iterations to assess the density and central tendency of the model’s predictive accuracy; **B** A comparative bar plot illustrating the mean performance values paired with their respective 95% confidence intervals to demonstrate statistical reliability; **C** Distribution box plots showing the median, quartiles, and range of the bootstrap results relative to the chance level (0.50) to highlight the consistency of the classification results; **D** A summary forest plot displaying the point estimates and 95% confidence intervals for AUC, accuracy, F1-score, precision, and recall, providing a consolidated view of the model’s statistical significance during external validation.

To assess the uncertainty and generalizability of model performance across diverse patient subgroups, we conducted bootstrap resampling and subgroup analyses on the external validation cohort. Bootstrap resampling with 1000 iterations yielded narrow 95% CIs for all key performance metrics, reflecting precise and stable estimates. The model achieved an AUC of 0.926 (95% CI: 0.889–0.961), demonstrating strong discriminative ability with minimal uncertainty, alongside an accuracy of 0.879 (95% CI: 0.840–0.918) and an F1-score of 0.800 (95% CI: 0.713–0.848) (Fig. 10). Further evaluation of robustness revealed that the bootstrap distributions for AUC, accuracy, F1-score, precision, and recall were approximately normal and tightly clustered well above the 0.5 chance baseline (Fig. 10A), indicating high internal consistency. A bar plot of the 95% CIs confirmed the AUC as the most stable metric, with a mean exceeding 0.85, followed by accuracy and precision (Fig. 10B). Box plots illustrated low variance and consistently high median performance (Fig. 10C). Finally, a forest plot summarizing the 95% CIs visually reinforced that the model’s predictive performance was statistically significant and resilient to sampling variability (Fig. 10D). Subgroup analysis was performed across clinically relevant strata defined by the 10 model features. The forest plot demonstrates that the model maintained high discriminative performance across all examined demographic and biochemical strata, with AUC values consistently exceeding 0.70. Notably, the model achieved exceptional diagnostic accuracy in the low C-reactive protein (CRP < 36.55 mg/L) subgroup, where the point estimate for AUC approached 0.98. High performance was also sustained in subgroups defined by elevated total bilirubin (TBIL ≥ 11.18 μmol/L), high basophil counts (≥0.030 × 10⁹/L), and high-density lipoprotein (HDL ≥ 0.93 mmol/L), all yielding mean AUCs above 0.90. While point estimates showed slight variation across age groups, gender, and calcium levels, the 95% confidence intervals remained well above the 0.50 chance threshold in every category, including traditionally complex cases with high inflammatory markers like ESR (≥35.64 mm/h) or low albumin (<36.90 g/L). Even in the subgroup with low neutrophil counts (<4.82 × 10⁹/L), which exhibited the widest confidence interval due to a smaller number of events, the model retained a mean AUC of ~0.73. These findings indicate that the predictive power of the routine blood biomarker-based model is robust and remains statistically significant regardless of the specific systemic or laboratory characteristics of the patient population (Supplementary Fig. 2 and Supplementary Table S2).

LRINEC performance was evaluated on the training set, internal validation set, and external validation set. Bootstrap resampling with 1000 iterations was performed for all three datasets to calculate 95% confidence intervals. The LRINEC score achieved comparable discriminative ability across all datasets, with AUC values ranging from 0.745 to 0.767. Bootstrap analysis revealed robust performance across datasets: Training [AUC 0.745 (0.719–0.771), Accuracy 0.826 (0.810–0.841), F1 0.521 (0.500–0.542)], Internal Validation [AUC 0.745 (0.691–0.800), Accuracy 0.831 (0.801–0.861), F1 0.542 (0.497–0.591)], External Validation [AUC 0.767 (0.687–0.838), Accuracy 0.817 (0.767–0.868), F1 0.470 (0.438–0.520)] (Supplementary Fig. 3). Compared to our 10-feature LightGBM model (external validation AUC 0.926 (95% CI: 0.889–0.961), the LRINEC score demonstrated substantially lower discriminative ability for differentiating NF from OM.

### Selected model interpretation

**Fig. 11:**
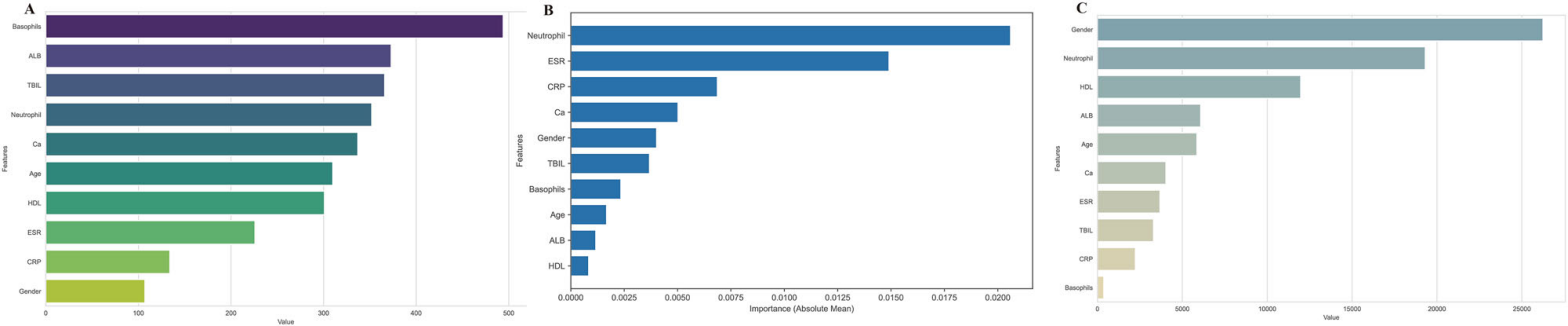
Global feature importance analysis of the optimized LGB model. **A** A horizontal bar chart illustrating the top ten features ranked according to the Gini importance (mean decrease in impurity) metric derived from the tree-based model structure; **B** A horizontal bar chart showing the top ten features ranked by permutation importance, which is calculated as the mean decrease in model performance on the external test set after randomly permuting the values of a single feature; **C** A horizontal bar chart depicting a global feature importance ranking generated by aggregating the absolute contributions of each feature from Local Interpretable Model-agnostic Explanations (LIME) across all samples in the external test set.

To elucidate the key drivers of the final model’s predictions, we employed multiple global feature importance techniques, which revealed consistent yet nuanced contributions from different biomarkers (Fig. 11). The Gini importance analysis identified basophil count as the single most influential feature by a substantial margin, followed by markers of metabolic and hepatic function such as ALB, TBIL, and Ca, as well as neutrophil count (Fig. 11A). In contrast, the permutation importance method, which assesses feature utility by measuring the drop in model performance upon feature shuffling, highlighted neutrophil count as the most critical predictor, followed by established inflammatory markers ESR and CRP (Fig. 11B). A third approach, aggregating local explanations from LIME into a global overview, presented another perspective, ranking Gender and neutrophil count as the top two most impactful features, with HDL cholesterol also showing high importance (Fig. 11C). While the exact ranking varied by method, a core set of features related to acute inflammation (neutrophil, CRP, ESR), metabolic derangement (ALB, Ca, HDL), and patient demographics (Age, Gender) were consistently implicated as primary drivers in differentiating necrotizing fasciitis from osteomyelitis.

**Fig. 12:**
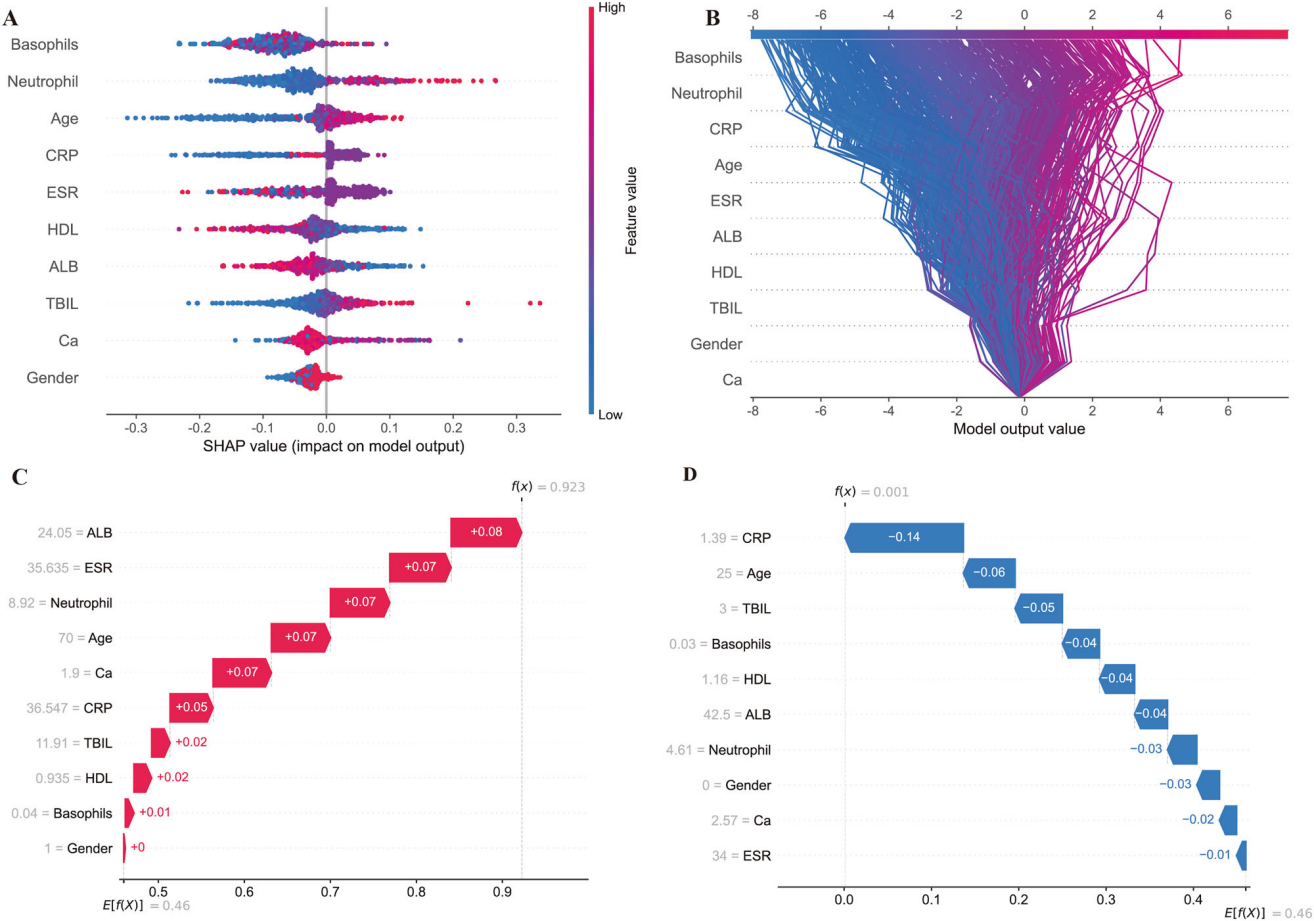
SHAP-based explainability of the optimized LGB model. **A** A SHAP summary plot combining feature importance with feature effects, where each point represents a patient for a given feature, its horizontal position indicates the SHAP value, and its color denotes the feature’s value from low (blue) to high (red); **B** A SHAP decision plot showing the model’s output value paths for the entire external test set, with each line representing an individual patient’s prediction as it is additively influenced by each feature; **C** A SHAP waterfall plot detailing the feature contributions for a single representative patient predicted to have necrotizing fasciitis, showing how each feature value shifts the prediction from the baseline expectation to the final output; **D** A corresponding SHAP waterfall plot for a representative patient predicted to have osteomyelitis.

The SHAP analysis provided comprehensive insights into feature contributions to the model’s predictive performance. The global SHAP summary plot revealed that high basophil and neutrophil counts were the most powerful predictors pushing the model’s output towards a diagnosis of NF, as indicated by the wide distribution of red points with positive SHAP values (Fig. 12A). This analysis also showed that higher age, CRP, and ESR, along with lower levels of HDL, ALB, and Ca, consistently increased the likelihood of an NF prediction. We also conducted a cross-model explainability analysis to verify the robustness of feature importance. SHAP summary plots were generated for other top-performing tuned models, as presented in Supplementary Fig. 4, which illustrates the consistent feature importance across different model architectures. Despite variations in models (LGB, GB, XGB, RF) and feature sets, a core group of biomarkers consistently emerged as the most significant predictors. Specifically, Neutrophil count, CRP, Age, Albumin, and Calcium were also almost universally ranked among the top contributors driving the model’s prediction towards NF. Furthermore, to assess interpretability stability across training centers, we compared SHAP feature importance rankings between the C1 model (trained on Center 1) and the C2 model (trained on Center 2). The analysis revealed a statistically significant moderate correlation between the two models’ feature importance patterns (Spearman ρ = 0.66, *P* = 0.04) (Supplementary Fig. 5 and Supplementary Table S3). Core inflammatory markers (Neutrophil, ESR and CRP) consistently remained within the top-nine features in both models, demonstrating robust interpretability stability across training centers rather than center-specific dependencies.

The decision plot further illustrated the cumulative impact of these features, showing how the prediction paths for individual patients diverged, with most NF cases (pink lines) being driven upwards by these key biomarkers (Fig. 12B). At the individual patient level, a waterfall plot for a representative NF case (final prediction score = 0.923) demonstrated how factors such as low albumin (24.05 g/L, SHAP value + 0.08), high ESR (35.6 mm/hr, SHAP value + 0.07), and high neutrophils (8.92 ×10⁹/L, SHAP value + 0.07) cumulatively drove the prediction far above the baseline risk (Fig. 12C). Conversely, for a patient with osteomyelitis (final prediction score = 0.001), the waterfall plot showed that low CRP (1.39 mg/L, SHAP value −0.14) and younger age (25 years, SHAP value −0.06) were the primary contributors that pushed the prediction significantly below the baseline, confirming the model’s reliance on clinically intuitive patterns (Fig. 12D).

### Clinical application online deployment

**Fig. 13:**
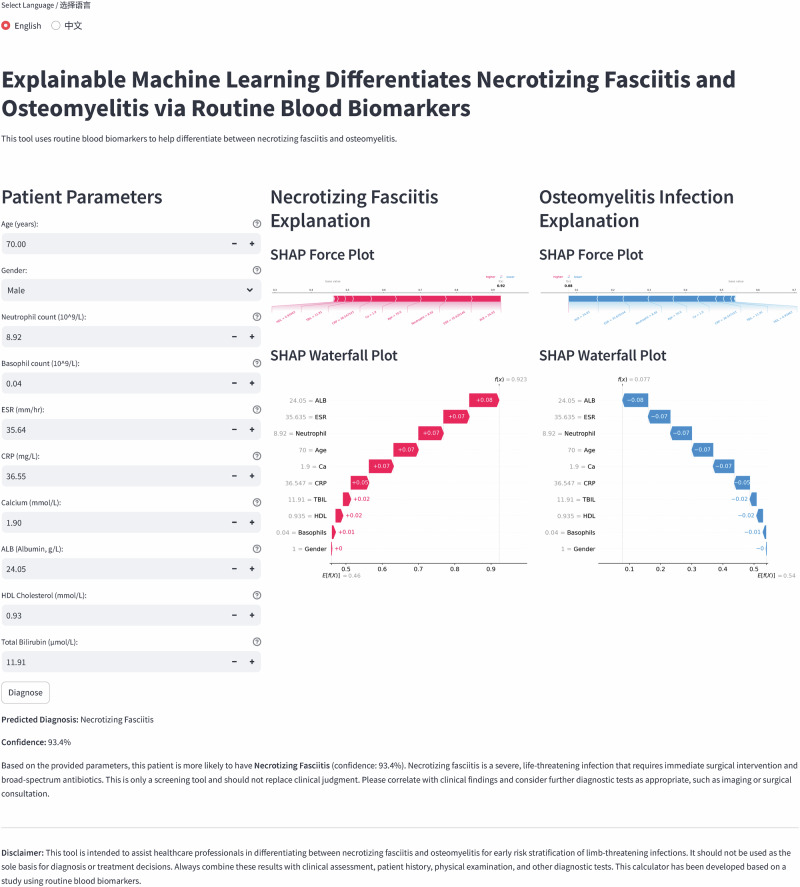
Web-based clinical decision support tool for differential diagnosis of necrotizing fasciitis and osteomyelitis in diabetic foot patients. Screenshot of the Flask-deployed interface allowing clinicians to input key features and receive real-time risk predictions. Integrated SHAP/LIME outputs provide case-level interpretability to support transparent clinical decision-making.

To translate our findings into a clinically actionable format, the final optimized model was successfully deployed as an interactive, web-based clinical decision support tool (Fig. 13), which is publicly accessible online (https://nf-osteo.streamlit.app/). The tool’s interface allows clinicians to input the ten routine biomarkers—age, gender, neutrophil and basophil counts, ESR, CRP, calcium, albumin, HDL, and total bilirubin—for a specific patient. Upon submission, the application provides an instantaneous differential diagnosis between NF and osteomyelitis, accompanied by a quantitative confidence score. For the example case shown, the tool predicted NF with 93.4% confidence based on the entered parameters. Crucially, to foster clinical trust and transparency, the tool simultaneously generates patient-specific SHAP force and waterfall plots in real-time. These visualizations quantitatively delineate how each biomarker contributed to the final prediction; for instance, the waterfall plot for the NF prediction clearly illustrates that low albumin (24.05 g/L) and high neutrophil count (8.92 ×10⁹/L) were the strongest drivers pushing the prediction towards NF. This integration of explainability, coupled with features like bilingual support and a prominent disclaimer, provides a tangible proof-of-concept for deploying responsible AI to augment decision-making in diagnostically challenging scenarios.

## Discussion

The burden of NF and OM was significant, as these severe, life-threatening infections contributed substantially to morbidity, mortality, and healthcare costs, particularly among vulnerable populations such as individuals with diabetes mellitus^14^. Although NF was relatively rare, it exhibited rapid progression and high mortality rates, with studies reporting mortality rates approaching 10%, and even higher among patients with comorbidities like diabetes^15^. Similarly, OM frequently occurred as a serious complication in patients with diabetic foot ulcers, often leading to limb amputation and death. The insidious onset and atypical clinical presentations of these infections frequently caused delays in diagnosis, which was a key factor contributing to poor outcomes and increased healthcare expenditures. The integration of explainable ML approaches with these routine biomarkers not only enhanced diagnostic accuracy but also provided transparency in decision-making, which was critical for clinical adoption and trust. Collectively, these findings supported the viability of explainable ML as a rapid, accessible, and effective strategy for early risk stratification and differentiation of limb-threatening infections such as NF and OM23. In this study, we tried to address the challenges posed by the diagnostic limitations of traditional methods and underscored the transformative potential of ML-driven solutions in improving patient outcomes and reducing healthcare burdens.

In this study, we developed and validated an explainable machine learning model capable of accurately differentiating necrotizing fasciitis from osteomyelitis using only ten routine blood biomarkers. Our expanded evaluation of resampling techniques confirmed SMOTE as the optimal method, yielding superior metrics compared to ROS and RUS, which highlighted the importance of synthetic oversampling in imbalanced clinical datasets. Our systematic evaluation of 110 unique feature selection and algorithm combinations revealed that gradient boosting approaches consistently delivered superior performance, with our parsimonious LightGBM model achieving exceptional discriminative ability on external validation (AUC = 0.921, 95% CI: 0.878–0.959). Notably, we observed that the AUC on the external test set (0.921 for the optimal 10-feature LGB model) was higher than on the internal test set (0.874–0.891 across top models). While external validation typically yields somewhat lower performance due to population differences, this pattern does not necessarily indicate overfitting or methodological artifact. Baseline characteristics comparison confirmed that the two cohorts exhibited highly similar patient demographics and clinical features across nearly all parameters, suggesting comparable case-mix between centers. To rigorously assess whether the observed performance pattern was dependent on arbitrary center allocation, we conducted a reciprocal validation experiment by training the model on Center 2 (*n* = 257) and testing on Center 1 (*n* = 3158). This reversed configuration maintained robust discriminative ability with an AUC of 0.840 (95% CI: 0.822–0.857)—significantly exceeding chance performance despite the markedly reduced training sample size. Critically, the preservation of clinically meaningful discrimination in both validation directions demonstrates that model performance was not an artifact of center-specific characteristics or allocation choices. The performance decrement observed in the reciprocal direction (0.840 vs. 0.921) is consistent with expectations given the pronounced imbalance in training set sizes, which inherently limits model complexity and generalization capacity when training on the smaller cohort. Collectively, these bidirectional validation results substantiate the model’s transportability across institutions within our regional healthcare system and indicate that the observed performance pattern likely reflects cohort-specific sampling variability rather than systematic bias or case-mix differences. This reciprocal analysis reinforces that the observed performance pattern stems from cohort characteristics rather than methodological artifact, and it underscores the model’s stability across different data configurations.

The robustness of our model was further supported by comprehensive stability and generalizability analyses. Bootstrap resampling with 1000 iterations on the external validation set yielded narrow 95% confidence intervals for all performance metrics, indicating precise performance estimates and minimal uncertainty in model performance. Evaluation across 10 different random seeds demonstrated excellent stability, with a coefficient of variation of only 0.4% for AUC, confirming that model performance is robust to stochastic variation in data splitting and resampling. Furthermore, subgroup analysis across clinically relevant strata defined by the 10 model features revealed consistent performance across diverse patient populations, with all subgroups achieving AUC values exceeding 0.70. This broad consistency across gender, age groups, and inflammatory marker levels supports the generalizability of our model across different patient demographics and disease presentations. Importantly, the features used for subgroup analysis were restricted to the 10 predictors in the final model, ensuring that the subgroup comparisons reflect performance on the same feature set used for primary analysis.

The model demonstrated robust generalizability across different patient populations and maintained strong performance metrics when subjected to threshold optimization, with decision curve analysis confirming its clinical utility across a wide range of threshold probabilities. Multiple explainability techniques, including SHAP and LIME analyses, revealed that the model’s predictions were primarily driven by clinically meaningful patterns of inflammatory markers (neutrophil count, ESR, CRP), metabolic derangements (low albumin, calcium, HDL), and demographic factors (age, gender), aligning with the established pathophysiology of these distinct infections. To facilitate clinical translation, we successfully deployed this model as a web-based decision support tool that provides real-time, interpretable predictions with visualization of feature contributions, offering a promising approach to early risk stratification in these limb-threatening conditions while maintaining appropriate transparency regarding the tool’s intended use as a supplement to clinical judgment. Our results demonstrated that ensemble-based machine learning models, particularly those leveraging L1-regularized feature selection in combination with algorithms such as Random Forest, Gradient Boosting, and LightGBM, consistently achieved high discriminative performance in differentiating NF from OM. The final model, based on a parsimonious set of ten routinely available biomarkers, achieved an AUC of 0.926 in external validation, with robust calibration and clinical utility as evidenced by decision curve analysis. These findings are consistent with prior literature highlighting the superior performance of ensemble methods in disease classification tasks, especially when compared to traditional linear models or single-feature scoring systems such as the LRINEC score^16^. Notably, our model outperformed LRINEC and its variants, which have shown limited sensitivity and specificity in both retrospective and prospective studies, often resulting in high false positive and false negative rates^17^. The clinical utility of our model is further supported by direct comparison with the established LRINEC clinical scoring system. LRINEC, a widely validated tool for distinguishing necrotizing fasciitis from other soft tissue infections, achieved an AUC of 0.767 (95% CI: 0.683–0.840) on our external validation cohort. Our 10-feature LightGBM model significantly outperformed LRINEC with an AUC of 0.926, representing better improvement in discriminatory ability. Importantly, the ML model achieved substantially better balance between sensitivity and specificity, addressing a critical limitation of LRINEC, which has relatively modest sensitivity. This performance advantage is particularly meaningful because LRINEC was developed for the broader indication of necrotizing soft tissue infections in general, whereas our model is specifically optimized for the challenging NF versus OM differential diagnosis. Despite the demonstrated statistical performance advantages of our model, we acknowledge important limitations regarding clinical impact validation. The retrospective nature of our study precludes assessment of real-world clinical utility, including impact on diagnostic delay, time to surgical intervention, rates of misclassification, or patient-centered outcomes such as mortality, limb salvage, or length of stay. A statistical advantage over established clinical scores does not necessarily translate to improved patient care or outcomes. Prospective interventional studies are essential next steps to determine whether incorporating our model into clinical decision-making actually changes physician behavior, improves diagnostic efficiency, reduces time to appropriate treatment, or ultimately leads to better patient outcomes compared to standard practice. The ultimate value of any clinical decision support tool depends not on its AUC, but on whether it helps clinicians make better decisions that improve patient lives. The inclusion of explainability techniques, such as SHAP and LIME, further enhanced the model’s transparency, allowing for both global and patient-specific interpretation of feature contributions—a critical factor for clinical adoption and trust^18^. Crucially, our cross-model SHAP analysis confirmed that the importance of key biomarkers—such as neutrophil count, C-reactive protein (CRP), and albumin—was not an artifact of the chosen algorithm (LightGBM), but a robust signal consistently observed across multiple top-performing models, thereby strengthening the biological plausibility of our findings.

A critical methodological consideration in developing machine learning models for tasks with inherent class imbalance, such as differentiating rare from common diseases, was the selection of an appropriate data balancing strategy. Our systematic comparative evaluation of eight resampling techniques demonstrated that this choice had a substantial and measurable impact on model performance, with the mean AUC varying by over 10 percentage points between the highest-performing (SMOTE: 0.843) and lowest-performing methods. This finding aligned with and extended prior literature in medical machine learning, which had often shown that sophisticated synthetic sampling methods could outperform naive random techniques. The superior performance of SMOTE in our study could be attributed to its ability to expand the minority class decision boundary by generating plausible synthetic samples through interpolation, thereby providing the model with richer information without the overfitting risks of simple replication or the information loss inherent to undersampling. Our observation that SMOTE achieved the best balance across multiple metrics—not just AUC but also accuracy and F1 score—was particularly relevant for clinical applications where both false negatives and false positives carried significant consequences. Interestingly, more complex variants of SMOTE did not uniformly outperform the standard implementation in our application, suggesting that the additional complexity introduced by hybrid cleaning steps or adaptive density weighting might not always provide incremental benefit and underscored the importance of empirical, task-specific evaluation. Our transparent reporting of this comprehensive resampling comparison provided valuable guidance for researchers developing models for similar diagnostic tasks and exemplified the principle that methodological rigor in medical machine learning required systematic, evidence-based justification of data preprocessing decisions.

The biological and pathophysiological overlap between NF and OM posed a significant diagnostic challenge, as both conditions could trigger marked elevations in inflammatory markers and metabolic disturbances due to severe infection. Kim et al. conducted a retrospective study on diabetic foot complications and reported that mean CRP levels were significantly higher in NF (12.9 ± 1.32 mg/dL) compared to OM (3.5 ± 0.41 mg/dL, *P* < 0.001). Additionally, albumin levels were notably lower in NF, consistent with our findings where NF patients exhibited a mean albumin of 31.2 g/L compared to 37.9 g/L in OM^19^. This pattern of pronounced hypoalbuminemia and elevated CRP in NF reflected the acute systemic inflammatory response and capillary leak syndrome characteristic of fulminant soft tissue infections. Similarly, our cohort demonstrated significantly higher ESR and neutrophil counts in NF, aligning with Kim et al.’s results and supporting the notion that while both diseases elevated these markers, the magnitude was greater in NF. Goh et al., in a systematic review, emphasized that both NF and OM could present with raised inflammatory markers but highlighted that the rapid progression and severity of laboratory derangements—such as marked leukocytosis and CRP elevation—were more characteristic of NF, consistent with our observation of a more severe systemic insult in NF patients^20^. Furthermore, Gore et al.’s study on odontogenic NF reported that hypoalbuminemia and metabolic derangements were common in severe cases and that comorbidities like diabetes mellitus significantly increased mortality risk, a finding that resonated with our data showing more profound metabolic dysfunction in NF and a higher prevalence of comorbidities^15^. Regarding metabolic derangements, both our study and prior literature documented that hypocalcemia was more pronounced in NF, likely due to extensive tissue destruction and systemic inflammation, whereas OM tended to present with milder metabolic abnormalities. The overlap in laboratory findings was further complicated by the fact that both conditions could induce systemic inflammatory response syndrome (SIRS), leading to similar elevations in CRP, ESR, and neutrophil counts, as well as reductions in albumin and calcium. However, the degree of these changes, consistently reported by Kim et al., Goh et al., and Gore et al., was typically more severe in NF, as reflected in our cohort’s data. The underlying pathophysiology involved widespread endothelial activation, increased vascular permeability, and a cytokine-driven catabolic state, which were more exaggerated in NF due to its rapid progression and extensive tissue involvement. This explained why, despite the overlap, extreme laboratory abnormalities—such as very high CRP, ESR, and neutrophil counts, coupled with marked hypoalbuminemia and hypocalcemia—should heighten clinical suspicion for NF over OM. In summary, our findings strongly agreed with previous studies, collectively demonstrating that while both NF and OM shared a biological profile of elevated inflammatory markers and metabolic derangements, the magnitude and rapidity of these changes were greater in NF, providing a critical, albeit nuanced, basis for differential diagnosis^15,19,20^.

The differences between NF and OM extended to changes in lipid indicators such as HDL and TBIL, both of which were sensitive to the systemic inflammatory response triggered by severe infection. In our study, we observed that NF patients exhibited significantly lower HDL and higher TBIL compared to OM patients, indicating more profound metabolic and hepatic dysfunction in NF. This pattern aligned with findings from several previous studies across various disease contexts. Chidambaram et al. conducted a retrospective cohort study in tuberculosis patients and reported that higher baseline HDL and TC were independently associated with lower CRP, WBC, and neutrophil-lymphocyte ratio (NL ratio), as well as reduced all-cause and infection-related mortality; conversely, lower HDL was linked to greater systemic inflammation, mirroring our observation that NF, as the more severe infection, was characterized by lower HDL and higher inflammatory markers^21^. Wei et al. analyzed over 13,000 patients with psychiatric disorders and demonstrated that lower HDL correlated with higher neutrophil counts and increased immune-inflammatory responses, reinforcing the link between inflammation and suppressed HDL, a relationship also evident in our NF cohort^22^. Song et al. investigated diabetic patients with peripheral arterial disease and found that the neutrophil/HDL ratio (NHR) was significantly higher in those with more severe disease and served as an independent risk factor for disease progression; this supported our finding that NF, associated with more severe systemic inflammation, also presented with lower HDL and higher neutrophil counts compared to OM^23^. Che et al., using UK Biobank data, showed that higher triglyceride/HDL-C ratios were associated with increased cardiovascular disease risk, largely mediated by dyslipidemia and systemic inflammation, further supporting the notion that lipid derangements serve as both markers and mediators of inflammatory disease severity^24^. Sacks et al. provided a nuanced perspective by analyzing HDL subspecies in four large prospective cohorts, finding that certain protein-defined HDL subspecies were associated with a higher risk of coronary heart disease, particularly those involved in inflammation and immunity, while others (such as those containing apoC1 or apoE) were protective; this suggested that not only the quantity but also the quality and composition of HDL may shift in severe infections like NF, potentially amplifying the observed differences with OM^25^. Compared to these studies, our results stood out because the reduction in HDL and elevation in TBIL were more pronounced in NF than in OM, likely reflecting the greater systemic inflammatory burden, hepatic stress, and tissue destruction in NF. The acute phase response to severe infection suppressed hepatic synthesis of HDL and impaired bilirubin clearance, leading to the observed laboratory changes. In summary, our findings were strongly supported by prior research: lower HDL and higher TBIL were consistently associated with more severe systemic inflammation and worse outcomes across a range of diseases, and in our cohort, these derangements were more marked in NF than in OM, offering a valuable diagnostic and prognostic distinction.

The study’s focus on model explainability directly addresses a central challenge in clinical AI: bridging the gap between high-performing algorithms and clinician trust. This approach is strongly supported by recent systematic reviews and empirical studies, which consistently highlight the necessity of transparent, interpretable models for clinical adoption and safe decision-making^26^. The use of SHAP, LIME, and permutation importance in our work mirrors best practices in the field. For example, Vimbi et al. systematically reviewed the application of LIME and SHAP in Alzheimer’s disease detection, concluding that these XAI frameworks are crucial for strengthening the trustworthiness of AI-based predictions and enhancing the fidelity of clinical decision support systems^27^. Similarly, Alabi et al. applied both SHAP and LIME to nasopharyngeal cancer survival models, finding that these techniques not only identified the most influential clinical features but also provided personalized, case-specific explanations that improved the understanding and confidence of clinicians in the model’s outputs^28^. Ghosh and Khandoker, in their investigation of chronic kidney disease prediction, demonstrated that SHAP and LIME could effectively visualize both global and local feature importance, with SHAP force plots offering individualized risk explanations and LIME clarifying the rationale behind specific predictions^29^. These findings are echoed in the broader medical AI literature, where Ali et al. reviewed 93 studies and found that the integration of XAI methods like SHAP and LIME consistently improved model transparency, interpretability, and ultimately, clinical trust^30^. In our study, the identification of acute inflammatory markers, metabolic indicators, and demographic factors as primary contributors aligns with established clinical knowledge and the results of prior machine learning research, reinforcing the model’s clinical plausibility and supporting its integration into practice. The nuanced differences in feature importance rankings across XAI methods, as observed in our analysis, are also well-documented in the literature and reflect the complex, multifactorial nature of infection-related laboratory changes^28,29^. Importantly, the deployment of our model as a web-based clinical decision support tool—with real-time, patient-specific explanations, bilingual support, and clear disclaimers—exemplifies responsible AI translation, as advocated by recent guidelines and retrospective studies^26,27^. Decision curve analysis in our work, demonstrating consistent net clinical benefit across a range of thresholds, further supports the model’s practical utility, echoing the recommendations that interpretable, evidence-based AI tools should augment, not replace clinical judgment in complex diagnostic scenarios. In summary, our approach and findings are strongly validated by systematic reviews and open-access retrospective studies, which collectively underscore the critical role of explainable AI in fostering trust, supporting decision-making, and enabling the responsible deployment of clinical decision support tools in healthcare. It is important to clarify the specific clinical scenarios in which our model is intended to be used. All patients in this study ultimately received a definitive diagnosis of NF or OM through standard clinical, imaging, microbiological, and histopathological work-up. In this context, the proposed model is not intended to replace the diagnostic gold standard, but rather to serve as an early risk stratification tool in the emergency department or inpatient evaluation of patients with suspected limb-threatening infections where the differential diagnosis between NF and OM is unclear. Specific clinical scenarios where the model may provide value include early presentation where physical examination findings are equivocal or atypical, particularly in patients with diabetes or other conditions that may mask classic symptoms; settings where MRI is not immediately available (after hours, resource-limited facilities, or centers without 24/7 MRI capability) and clinicians must make decisions about urgency of specialist consultation or surgical intervention; triage of patients to identify high-risk NF cases requiring urgent surgical escalation versus lower-risk cases that can be managed conservatively while awaiting definitive diagnostics; and support for less experienced clinicians or in settings with limited specialist availability by providing objective, data-driven probability estimates. We acknowledge that we have not quantified the impact of our model on important clinical endpoints such as diagnostic delay, time to surgery, rates of misclassification, or patient outcomes. While we demonstrate that the model can statistically distinguish NF from OM using routine blood biomarkers, prospective interventional studies are needed to determine whether using this tool actually improves patient care compared to standard practice. The value of a decision support tool ultimately depends on whether it leads to better clinical decisions and outcomes, not just on its statistical performance. This is an important limitation and an essential direction for future research.

Beyond demonstrating that our final model’s predictions were explainable and clinically interpretable, we sought to validate that the identified feature importance patterns represented robust, reproducible signals rather than artifacts of a specific algorithmic or methodological choice. This distinction was critical for establishing clinical trust in AI-based decision support tools. Our comprehensive cross-model SHAP validation analysis, which compared feature importance across nine distinct combinations of algorithms and feature selection strategies, revealed remarkable convergence on a consistent core set of biomarkers. The consistency with which neutrophil count, inflammatory markers (CRP, ESR), metabolic indicators (albumin, calcium, HDL), and demographic factors (age, gender) emerged as primary predictors—despite fundamental differences in how these models learned and represented data—provided strong evidence that these patterns were highly coherent with the underlying pathophysiological differences between necrotizing fasciitis and osteomyelitis. This finding was analogous to the scientific principle of reproducibility; just as a medical phenomenon gained credibility when confirmed by independent experiments, a model’s feature importance was more trustworthy when independently identified by diverse algorithms. This validation directly addressed a frequently-cited barrier to clinical AI adoption: the concern that models might base decisions on inscrutable or unstable patterns. By demonstrating that our model’s reliance on markers of severe inflammation and profound metabolic dysfunction was reproducible across diverse modeling architectures, we strengthened the case for its clinical trustworthiness and demonstrated a practical framework for responsible AI validation. Furthermore, the biological coherence of these consensus features with established NF pathophysiology provided external validation that the machine learning approach had identified medically meaningful patterns. While prior studies employing XAI in medicine had often focused on single-model explainability, our work extended this principle by demonstrating reproducibility across algorithmic diversity, thereby providing stronger evidence for the reliability and generalizability of the identified biomarker patterns. This had important practical implications, suggesting that the core feature importance structure might remain stable even if the model’s algorithm was updated in the future. In summary, the cross-model SHAP validation analysis substantially enhanced the credibility of our explainable AI framework by showing that local interpretability was complemented by the global reproducibility of feature importance patterns, collectively addressing key barriers to AI adoption in high-stakes clinical decision-making. Furthermore, our cross-center SHAP analysis demonstrated statistically significant moderate correlation (*Spearman ρ* = 0.66, *P* = 0.04) between feature importance rankings of models trained on different center datasets. Despite the moderate correlation strength, the statistical significance and consistency of core inflammatory markers (Neutrophil, CRP, ESR) provide important evidence that our model relies on consistent predictive patterns across institutions, rather than achieving comparable performance through center-specific feature dependencies^31,32^. This interpretability stability substantially strengthens our claims of clinical trustworthiness and model robustness in multi-center settings^33^. The moderate strength of the correlation (*ρ* = 0.66) primarily reflects the substantial 10-fold difference in training sample sizes between centers. Smaller training datasets may not capture the full spectrum of feature interactions, which can affect learned importance hierarchies^34,35^. However, the achievement of statistical significance (*P* = 0.04) despite this size disparity is encouraging and suggests that larger, more balanced multi-center cohorts would likely demonstrate even stronger interpretability- consistency.

Our study demonstrated several limitations that warrant careful consideration. First, the retrospective nature of the design, while enabling the analysis of a large cohort, inherently introduced a risk of selection bias and unmeasured confounding variables. As a result, we were able to identify strong associations but could not establish causality. Second, although we performed external validation on an independent cohort from a second center, both participating institutions are located within the same geographic region (Xinjiang Uyghur Autonomous Region, China) and healthcare system. This regional homogeneity limits the evidence for geographic, ethnic, and healthcare-system diversity that would ideally be demonstrated in a multicenter study. The model’s performance may vary when applied to populations from different geographic regions, ethnic backgrounds, or healthcare systems with different laboratory platforms, measurement techniques, and clinical pathways. While our study demonstrates the model’s ability to generalize across different institutions within our region, further validation in more diverse settings is needed before broader clinical deployment. The external validation cohort, while independent, had a relatively small sample size (*N* = 257), which may limit the precision of performance estimates. Third, the model relied solely on routine blood biomarkers collected within the initial 48 h of assessment, excluding clinical signs, comorbidities, or imaging data, which are critical components of a comprehensive diagnostic evaluation. While this approach enhanced the model’s simplicity and accessibility, its performance in conjunction with these additional clinical factors remains unexplored. Our subgroup analysis employed median dichotomization to stratify continuous variables, which has several important limitations that warrant acknowledgment. As highlighted in recent methodological literature^36,37^, median dichotomization may reduce statistical power compared to treating variables as continuous, potentially missing subtle effects. It may also obscure non-linear relationships between the predictor and outcome, such as threshold effects or U-shaped associations. Furthermore, the median value is sample-specific and may not generalize to other populations with different demographic or clinical characteristics. The approach creates an artificial boundary that may not reflect true biological thresholds, potentially misclassifying patients near the cutpoint. Future studies should consider more sophisticated approaches to strengthen subgroup analysis and provide deeper insights into effect heterogeneity. These could include using formal interaction testing with continuous variables to evaluate effect heterogeneity without dichotomization, applying clinically meaningful cutoff values based on established diagnostic or prognostic thresholds from clinical guidelines, or employing flexible modeling approaches such as restricted cubic splines or generalized additive models to capture non-linear relationships without arbitrary dichotomization. However, these approaches would require substantially larger cohorts to achieve adequate statistical power for reliable subgroup effect estimation^38^. Finally, the web-based tool served as a proof-of-concept for clinical translation; however, its real-world clinical utility and potential impact on patient outcomes have not been evaluated in a prospective, interventional trial. Future prospective studies are necessary to validate its performance and assess its integration into clinical workflows. These considerations highlight the need for further research to address the identified gaps and refine the model’s applicability across diverse settings.

Looking forward, several directions for future research emerge from this work. First, direct comparison of our model against established clinical scoring systems such as the LRINEC score would help quantify the incremental value of the machine learning approach over traditional methods. While our model demonstrates high discrimination, head-to-head comparative studies are needed to establish superiority or equivalence in real-world clinical settings. Second, prospective interventional trials are essential to determine whether using our model actually improves patient outcomes compared to standard care. Important endpoints would include time to definitive diagnosis, time to surgical intervention, rates of misclassification, amputation rates, mortality, and length of hospital stay. Third, expanding validation to include centers from different geographic regions, healthcare systems, and patient populations would provide stronger evidence for generalizability and help identify potential performance variations across different settings. Fourth, more comprehensive robustness analyses are needed, including systematic assessment of SMOTE synthetic sample clinical plausibility through dimensionality reduction visualizations, bootstrap confidence intervals for all performance metrics, subgroup analyses where sample sizes permit, and evaluation of model stability across different random seeds and data splits. Fifth, exploration of the model’s performance in different clinical scenarios—such as patients with diabetic foot ulcers, postoperative infections, or those presenting at different disease stages—would help clarify the specific use cases where the tool provides the most value. We acknowledge that as researchers still developing expertise in machine learning methodology, some of these advanced analyses require resources and expertise beyond our current capacity. However, we believe this work provides a foundation that can be built upon by the research community to achieve even greater rigor and clinical impact.

In conclusion, we developed, rigorously validated, and deployed a parsimonious, explainable machine learning model that accurately differentiates necrotizing fasciitis from osteomyelitis using only ten routinely available blood biomarkers. The model demonstrated excellent and generalizable performance, and its decisions are driven by clinically intuitive pathophysiological patterns. By translating this model into an accessible web-based tool with real-time, patient-specific explanations, we provide a promising, evidence-based instrument to augment clinical judgment, assist in early risk stratification, and potentially expedite life-saving interventions for patients with limb-threatening infections.

## Methods

This study adhered to the principles outlined in the Helsinki Declaration and received approval from the Ethics Committee of The Sixth Affiliated Hospital of Xinjiang Medical University and The First People’s Hospital of Kashi Prefecture. The requirement for informed consent was waived by the Ethical Committee, as the study involved de-identified data, posing no potential risk to patients and maintaining no connection between the patients and researchers. All procedures were conducted in compliance with the applicable guidelines and regulations. No biological specimens were used in this study.

### Study design

This retrospective multi-center study was conducted following approval from the Institutional Ethics Committees of the Sixth Affiliated Hospital of Xinjiang Medical University and The First People’s Hospital of Kashi Prefecture (designated Center 1 and Center 2, respectively). Adhering to the principles of the Declaration of Helsinki and relevant institutional guidelines, the committees waived the need for individual patient informed consent due to the retrospective nature of the investigation and the complete anonymization of patient data prior to analysis. The study aimed to develop and validate machine learning models for the early differentiation of necrotizing fasciitis (NF) from osteomyelitis (OM) in patients presenting with limb-threatening infections, using only routinely collected blood biomarkers.

### Patient cohort definition and data acquisition

We identified patients admitted to Center 1 (The Sixth Affiliated Hospital of Xinjiang Medical University) and Center 2 (The First People’s Hospital of Kashi Prefecture) between January 2014 and December 2024 with suspected limb-threatening infections requiring differentiation between NF and OM. Center 1 was designated as the development cohort due to its substantially larger sample size (*N* = 3158), which provided sufficient statistical power for robust model training and internal validation; its data were randomly divided, with 75% allocated to the training set and 25% reserved as an internal validation set. Center 2, as an independent institution within the same regional healthcare system but with distinct patient referral patterns and geographic location, served as the external validation cohort (*N* = 257) to assess model generalizability across institutions.

Inclusion criteria for patient enrollment were: (1) age 18 years or older; (2) presentation to one of the participating centers with clinical suspicion of a limb-threatening infection potentially representing NF or OM, including clinical presentation of acute or subacute limb-threatening infection with suspicion for necrotizing fasciitis (NF) or osteomyelitis (OM), as documented by treating clinicians; (3) availability of comprehensive routine blood biomarker results obtained within the initial 48 h of assessment, encompassing availability of complete blood biomarker data—including, but not limited to, white blood cell count, C-reactive protein (CRP), erythrocyte sedimentation rate (ESR), and procalcitonin—collected within 24 h of initial hospital evaluation; and (4) a definitive final diagnosis discriminating between NF and OM, established using a composite reference standard, which integrated clinical evaluation (e.g., physical examination findings suggestive of NF, probe-to-bone test results for OM), laboratory data, imaging findings (particularly Magnetic Resonance Imaging [MRI] where available), microbiological or histopathological confirmation from surgical debridement or biopsy when performed, and the documented clinical course including response to specific therapies, while also requiring concordant evidence from clinical assessment, imaging (such as MRI or CT), microbiological culture, and/or histopathological examination prior to definitive therapy^7,39,40^.

Exclusion criteria were: (1) age under 18 years; (2) primary limb pathology confirmed as non-infectious (e.g., acute major trauma, non-infected Charcot neuroarthropathy, crystalline arthropathy), or absence of limb-threatening infection or clinical suspicion for NF or OM; (3) presence of concurrent systemic inflammatory or infectious conditions potentially confounding biomarker interpretation (e.g., active autoimmune disease, sepsis from another source, recent major surgery, active malignancy under treatment), including presence of confounding conditions known to alter biomarker profiles or clinical presentation such as active autoimmune disease, systemic sepsis unrelated to limb infection, or concurrent malignancy; (4) incomplete core routine blood biomarker data (defined as >30% missing values for investigated features), or incomplete or missing blood biomarker data within the specified 24-h window; and (5) insufficient clinical, imaging, laboratory, or follow-up data to reliably classify the patient as having NF or OM according to the reference standard, including lack of diagnostic confirmation by the composite reference standard or ambiguous or indeterminate diagnoses where NF or OM cannot be reliably established, as well as alternative limb pathologies such as acute trauma, Charcot arthropathy, or chronic non-infectious inflammatory conditions, and insufficient clinical documentation or follow-up to confirm diagnostic outcomes. The patient selection process is detailed in Fig. 1.

The final diagnosis of NF versus OM was established through a comprehensive diagnostic process integrating multiple lines of evidence. All patients underwent detailed clinical assessment by attending spine surgeons or orthopedic specialists. Physical examination findings characteristic of NF included crepitus, bullae, skin necrosis, and positive ‘finger test’ (gas in tissues on palpation), whereas OM was suggested by localized bone tenderness, chronic wound characteristics, and signs of chronic infection^3,7^. Radiological imaging including radiographs, MRI, and in some cases CT or ultrasound was reviewed. MRI findings suggestive of NF included fascial thickening (≥3 mm), fluid collection along fascial planes, and absence of bone involvement^2^, while OM was diagnosed based on bone marrow edema, cortical destruction, or abscess formation adjacent to bone on MRI^9^. When surgical intervention was performed (debridement, resection, or amputation), tissue samples were sent for microbiological culture and histopathological examination. The presence of necrotic fascia with inflammatory infiltrate and positive deep tissue cultures supported NF diagnosis, while bone biopsy showing osteomyelitis confirmed OM. The response to treatment and clinical outcome over the follow-up period were also considered. Patients requiring extensive debridement or amputation with findings consistent with NF were classified accordingly, while those responding to long-term antibiotic therapy for bone infection were classified as OM. For cases where clinical, imaging, and pathological findings were discordant or inconclusive, the final diagnosis was determined through multidisciplinary discussion involving spine surgeons, radiologists, pathologists, and infectious disease specialists at each center. The consensus diagnosis from multidisciplinary review was used as the final classification. The machine learning analysis was conducted retrospectively using de-identified data. Clinicians who established final diagnoses during routine patient care were not blinded to laboratory values, as these were part of standard diagnostic workup. However, data scientists performing the machine learning analysis were not involved in the diagnostic process.

### Blood-based biomarker collection and data preprocessing

We collected demographic data (age, gender) and a panel of routine blood biomarkers typically assessed during the initial diagnostic workup for severe limb infections. These candidate features encompassed markers related to inflammation (e.g., white blood cell count [WBC] and differential, erythrocyte sedimentation rate [ESR], C-reactive protein [CRP]), renal function (e.g., creatinine, estimated glomerular filtration rate [eGFR], uric acid, cystatin C [CYS_C]), hepatic function (e.g., albumin [ALB], alanine aminotransferase [ALT], aspartate aminotransferase [AST], alkaline phosphatase [ALP]), electrolytes, muscle enzymes (creatine kinase [CK]), and lipid profiles (e.g., triglycerides [TG], high-density lipoprotein [HDL], low-density lipoprotein [LDL]).

Prior to model development, the data underwent preprocessing. Missing values within the biomarker dataset were imputed using the Random Forest imputation method^16^, leveraging relationships between features to estimate missing entries. Subsequently, all biomarker features were normalized using the *MinMaxScaler* to transform values into a uniform [0, 1] range, mitigating potential bias from differing measurement scales. To address the class imbalance between NF and OM cases in the training cohort, we conducted a systematic evaluation to identify the optimal data balancing strategy. We compared eight resampling methods: Random Oversampling (ROS), Random Undersampling (RUS), and several advanced hybrid techniques—including the Synthetic Minority Oversampling Technique (SMOTE), SMOTE combined with Edited Nearest Neighbors (SMOTE-ENN), SMOTE with Tomek links (SMOTE-Tomek), Adaptive Synthetic Sampling (ADASYN), KMeans-SMOTE, and SVM-SMOTE—all implemented with their default settings. The performance of each method was assessed by averaging the results of eleven different machine learning classifiers within a 5-fold cross-validation framework on the training data. The evaluation metrics included Area Under the Receiver Operating Characteristic Curve (ROC-AUC), accuracy, F1-score, precision, and recall. This technique generated synthetic samples of the minority class (potentially NF or OM, depending on prevalence) within the feature space, creating a balanced dataset for model training and reducing bias towards the majority class^41^. To further validate the clinical plausibility of SMOTE-generated synthetic samples and assess the robustness of our findings, we performed three complementary analyses. We employed low-dimensional dimensionality reduction techniques (principal component analysis [PCA] and t-distributed stochastic neighbor embedding [t-SNE]) to visualize the distribution of SMOTE-generated synthetic samples relative to real samples in feature space. PCA was applied to all biomarker features to reduce dimensionality while preserving variance, and the first two principal components were plotted to visualize class separation. t-SNE was applied with perplexity = 30 and n_iter = 1000 to capture non-linear patterns in the data.

### Competitive machine learning model development

To identify the optimal predictive signature for differentiating NF from OM, we implemented a comprehensive and systematic two-stage modeling strategy. This approach involved a competitive evaluation of ten distinct feature selection methods paired with a diverse panel of eleven machine learning algorithms, culminating in the development and validation of 110 unique candidate models pipeline, which contained features selection algorithm and classifier algorithm. In the first stage, we conducted an exhaustive feature selection process on the preprocessed training data to identify the most parsimonious and diagnostically relevant subset of biomarkers. To ensure robustness and mitigate method-specific biases, we employed a multi-pronged approach that included: (i) filter methods, utilizing univariate statistical tests to select the top 10, 15, and 20 features (SelectK10, SelectK15, SelectK20); (ii) wrapper methods, which use Recursive Feature Elimination (RFE) guided by both a Random Forest (RFE-RF) and a Logistic Regression (RFE-LR) model; and (iii) embedded methods, which perform feature selection as an integral part of the model training process, including L1-regularized Logistic Regression (L1-based), Random Forest (Tree-based), Gradient Boosting (GB-based), and Extra Trees (ET-based). A baseline scenario using all available features was also included, resulting in ten distinct feature subsets for downstream modeling.

**Fig. 14:**
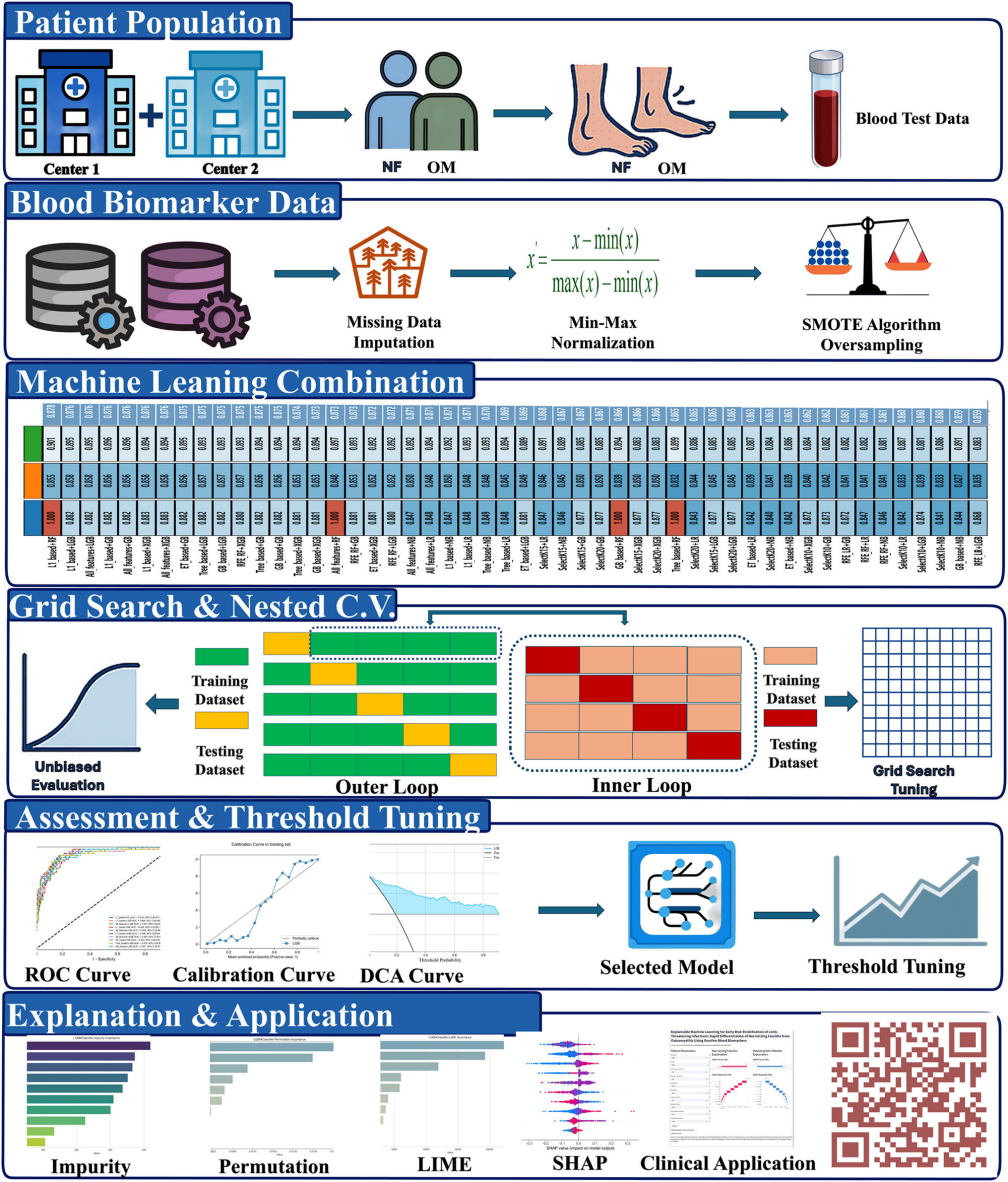
Comprehensive machine learning research workflow from data curation to clinical deployment. Schematic overview of the end-to-end pipeline, from data preprocessing and feature selection through nested cross-validation, model evaluation and application. The workflow emphasizes external validation and clinical translation via a web-based deployment framework.

In the second stage, each of the ten feature subsets was used to train a panel of eleven supervised machine learning classifiers. The algorithms were selected to span a wide range of machine learning families, including tree-based ensembles (Random Forest [RF], Gradient Boosting [GB], Extra Trees [ET], XGBoost [XGB], LightGBM [LGB]), a single Decision Tree (DT), linear models (Logistic Regression [LR], ElasticNet [EN]), a kernel-based method (Support Vector Classifier [SVC]), an instance-based method (K-Nearest Neighbors [KNN]), and a probabilistic classifier (Gaussian Naive Bayes [GNB]). This combinatorial strategy allowed for an unbiased, head-to-head comparison of 110 unique feature-algorithm pipelines. Each pipeline, which integrated feature scaling, selection, and classification, was trained on the training cohort. The definitive model was selected based on the highest average ROC-AUC across both the internal and external validation cohorts, ensuring that the final chosen model demonstrated not only superior predictive accuracy but also robust generalizability. Detailed information could be obtained from Fig. 14. Then, top ten algorithm combinations (Feature Selection + Classifier) were optimized by tuning. Model hyperparameters were optimized using nested cross-validation (5-fold outer loop, 4-fold inner loop) combined with grid search exclusively on the training data to maximize performance while minimizing overfitting risk^42^. We also conducted sensitivity analyses comparing four different class imbalance handling strategies: SMOTE oversampling, class-weighted loss functions, random under-sampling, and no adjustment. Model stability was evaluated across 10 different random seeds (42, 123, 456, 789, 2024, 999, 111, 222, 333, 444) to assess the impact of stochastic variation in data splitting and resampling on model performance and feature importance rankings. For each random seed, we calculated the coefficient of variation (CV %) for all performance metrics.

### Threshold tuning and model explanation

The performance of the developed models was rigorously evaluated on both the held-out internal testing set (from Center 1) and the independent external testing cohort (Center 2). Key discrimination metrics calculated included the area under the receiver operating characteristic curve (AUC), sensitivity, specificity, F1-score, accuracy, and balanced accuracy. Model calibration was assessed using the Brier score and log loss. An optimal probability threshold for clinical decision-making for the selected model, balancing the trade-off between sensitivity and specificity, was determined using the *TunedThresholdClassifierCV* method (5-fold cross-validation, 50 repeats) by optimizing for the F1-score^43^. Threshold optimization was performed exclusively on the Center 1 training dataset, with no information from the Center 1 internal test set or the Center 2 external validation cohort used during this process^44^. The optimal threshold was fixed after training and applied uniformly to the Center 2 external validation cohort, with no threshold re-optimization performed. This strict separation between training (including threshold tuning) and evaluation ensures that our reported performance metrics represent unbiased estimates of model generalizability^45^. To quantify uncertainty in model performance estimates and evaluate generalizability across patient subgroups, we performed two complementary analyses. First, bootstrap resampling was applied to the external validation set with 1000 iterations to calculate 95% confidence intervals for all performance metrics. Bootstrap provides non-parametric estimates of uncertainty without assuming specific distributions for the metrics, representing an efficient approach for internal validation that exploits all available data. Second, subgroup analysis was performed across clinically relevant strata defined by the model features. For each continuous feature, subgroups were defined using median split; for categorical features, subgroups were defined by the actual categories. Model performance was evaluated separately within each subgroup to assess consistency across diverse patient populations. Continuous variables were divided into two groups using the median value as the cutoff point. This approach was selected because median split provides balanced sample sizes in each subgroup, ensuring adequate statistical power for comparison, and creates clinically interpretable patient strata that can be easily communicated to clinicians without requiring specialized statistical knowledge.

To benchmark our model against established clinical scoring systems, we calculated the Laboratory Risk Indicator for Necrotizing Fasciitis (LRINEC) score for all patients using available biomarkers from our dataset. The LRINEC score incorporates six components: C-reactive protein (CRP), white blood cell count (WBC), hemoglobin, serum sodium, serum creatinine, and glucose^46^. Each component is assigned points based on laboratory values, with a total score ranging from 0 to 13. To contextualize our model’s performance within established clinical scoring frameworks, we evaluated the LRINEC score across all datasets. LRINEC scores were calculated using five of six standard components (C-reactive protein, white blood cell count, hemoglobin, sodium, and creatinine), as glucose measurements were not available. Each biomarker was assigned points according to validated criteria: CRP > 150 mg/L (4 points), WBC 15–25 × 10³/µL (1 point) or >25 × 10³/µL (2 points), hemoglobin 11–13.5 g/dL (1 point) or <11 g/dL (2 points), sodium <135 mmol/L (2 points), and creatinine >1.6 mg/dL (2 points). LRINEC Risk Stratification: Score ≤5 indicates low risk (NF probability <50%), Score 6–7 indicates medium risk (NF probability 50–75%), Score ≥8 indicates high risk (NF probability >75%). Due to missing glucose values in our dataset, LRINEC scores were also calculated using the five available components (CRP, WBC, hemoglobin, sodium, and creatinine).

To enhance clinical trust and provide insights into model decision-making, we employed explainable AI (XAI) techniques. SHapley Additive exPlanations (SHAP) values were computed to quantify the contribution of each selected biomarker to the model’s prediction for individual patients (local interpretability) and across the cohort (global feature importance)^47^. As a complementary approach, Local Interpretable Model-agnostic Explanations (LIME) was used to generate alternative local explanations by approximating the complex model’s behavior around specific predictions with simpler, interpretable models^48^. These analyses aimed to identify the key biomarkers driving the differentiation between NF and OM.

### Development of a clinical decision support tool

To facilitate the translation of our model into a clinically accessible format, we developed a prototype web-based clinical decision support tool. This application was built using Python and the *Streamlit* framework, an open-source library tailored for rapid prototyping of data science applications. The previously trained and optimized model, along with its corresponding SHAP explainer object, was serialized using the *joblib* and *pickle* packages, respectively, and subsequently loaded into the application’s backend. The user interface was designed to request ten specific input features utilized by the model. Simultaneously, the SHAP explainer computed patient-specific Shapley values in real-time. These values were then programmatically displayed as both force plots and waterfall plots, offering a quantitative and visual explanation of each biomarker’s contribution to the model’s output for the specific case. For responsible deployment, the interface included a disclaimer emphasizing its role as an assistive tool and provided context-specific guidance based on the predictive output. Multilingual support was also integrated to enhance usability across diverse clinical settings.

### Statistical analysis

Descriptive statistics were used to summarize patient baseline characteristics. Continuous variables were assessed for normality using visual inspection of histograms and Q-Q plots. Normally distributed continuous variables are presented as mean ± standard deviation (SD), while non-normally distributed variables are presented as median with interquartile range (IQR). Group comparisons were performed using the Student’s *t* test for normally distributed variables and the Mann–Whitney U test for non-normally distributed variables. Categorical variables, presented as counts and percentages (%), were compared using the Chi-square test or Fisher’s exact test, as appropriate. To evaluate cross-center interpretability stability, *Spearman* rank correlation analysis was performed to compare SHAP feature importance rankings between models trained on different centers. A two-sided *P*-value < 0.05 was considered statistically significant. All baseline statistical analyses were conducted using R (version 4.2.1). Machine learning model development, feature selection, performance evaluation, and XAI analyses were performed using Python (version 3.9.5) with libraries including Scikit-learn, XGBoost, LightGBM, SHAP, LIME, and Streamlit.

## Supplementary information

Supplementary Materials

## Data Availability

The source data for all models, tables, and figures, along with supporting materials, are available from the corresponding author upon reasonable request. The source code generated for this study is not publicly released to safeguard proprietary intellectual property. However, academic researchers affiliated with recognized institutions may request access to the codebase—including data preprocessing pipelines, model architecture specifications, and training procedures—for noncommercial research purposes. Such requests should be directed to the corresponding author, Xinghua Song (songxinghua19@163.com), and must be accompanied by a clear and detailed research proposal describing the intended application. All requests will undergo review by the institutional oversight committee, with decisions typically communicated within 4–6 weeks. Approved users will be required to sign a formal usage agreement that explicitly prohibits redistribution, commercial use, or incorporation into proprietary systems.
