# Supplementary material for: Explainable machine learning differentiates necrotizing fasciitis and osteomyelitis via routine blood biomarkers
Source: NPJ Digit Med. 2026 Apr 29;9:507. doi: 10.1038/s41746-026-02686-3 (PMC13333845; doi:10.1038/s41746-026-02686-3)
Supplement: Supplementary file 1 — Supplementary Materials [file 41746_2026_2686_MOESM1_ESM.pdf]

Explainable Machine Learning Differentiates Necrotizing Fasciitis and Osteomyelitis via Routine Blood Biomarkers

Supplementary Files

Supplementary Table 1 Baseline Comparison by Center

Variable	Center 1 (n=3158)	Center 2 (n=257)	*P*-value
Age	44.75 ± 18.24	44.58 ± 19.02	0.8441
Gender: Female	996 (31.5%)	83 (32.3%)	1.0000
Gender: Male	2162 (68.5%)	174 (67.7%)	
WBC	9.07 ± 5.23	9.03 ± 5.47	0.4721
Neutrophil	6.20 ± 4.80	6.17 ± 5.40	0.3274
Monocyte	0.67 ± 0.85	0.69 ± 0.92	0.5508
Basophils	0.03 ± 0.02	0.03 ± 0.02	0.8465
Eosinophils	0.15 ± 0.14	0.16 ± 0.17	0.7679
ESR	35.63 ± 14.52	35.64 ± 13.21	0.9507
CRP	36.80 ± 36.17	33.47 ± 34.45	0.0692
ALB	36.77 ± 6.42	36.85 ± 6.58	0.6759
Ca	2.04 ± 0.47	2.02 ± 0.49	0.6220
HDL	0.93 ± 0.29	0.95 ± 0.31	0.7742
TBIL	12.89 ± 14.58	11.25 ± 5.80	0.1882
TC	3.74 ± 0.81	3.69 ± 0.89	0.0922
TG	1.40 ± 0.86	1.35 ± 0.94	0.0582
K	3.85 ± 0.55	3.84 ± 0.47	0.8048
Na	138.43 ± 4.79	138.58 ± 4.00	0.6480
Cl	103.61 ± 4.92	103.58 ± 3.85	0.9521
Mg	0.85 ± 0.10	0.84 ± 0.09	0.1196
P	1.27 ± 0.32	1.28 ± 0.32	0.1101
Creatinine	73.25 ± 129.17	66.52 ± 66.71	0.5318
eGFR	110.18 ± 18.19	110.02 ± 14.63	0.9474
Uacid	279.89 ± 165.06	291.68 ± 196.23	0.7939
CYS_C	0.92 ± 0.37	0.90 ± 0.22	0.8735
UMAlb	353.29 ± 85.77	349.13 ± 73.06	0.0504
UACR	628.21 ± 64.55	627.49 ± 33.31	0.6653
HbA1c	7.98±1.39	7.96±1.36	0.8689
TP	69.57 ± 8.48	69.13 ± 8.31	0.4698
AST	32.52 ± 62.64	26.64 ± 20.43	0.0029
ALT	29.60 ± 51.93	23.24 ± 19.40	0.0004
AFU	19.96 ± 5.10	20.12 ± 7.01	0.7642
NT_5	6.00 ± 3.71	5.75 ± 2.66	0.2653
LDL	2.42 ± 0.64	2.38 ± 0.70	0.1912
GGT	42.18 ± 49.84	36.88 ± 39.14	0.0770
ALP	118.73 ± 72.00	123.65 ± 65.60	0.1613
LDH	253.78 ± 703.99	217.03 ± 180.89	0.0853
DBIL	2.27 ± 7.68	2.00 ± 3.18	0.3261
IBIL	7.00 ± 6.12	6.34 ± 4.09	0.3895
HB	120.65 ± 23.85	118.19 ± 23.05	0.0748
Hematocrit	35.80 ± 9.55	35.51 ± 8.66	0.1968
Platelet	313.82 ± 128.38	324.15 ± 137.58	0.4621

CK	156.78 ± 617.09	105.03 ± 96.62	0.0985
CK_MB	16.86 ± 8.68	16.72 ± 4.33	0.7379

In Supplementary Table 1, all continuous variables are presented as mean ± standard deviation (SD), implying an assumption of approximate normality, while categorical variables are expressed as counts with percentages (n (%)). The full names and units of the variables are as follows: Age (years); Gender (Female/Male); WBC – white blood cell count ($\times 10^9$/L); Lymphocyt – lymphocyte count ($\times 10^9$/L); Neutrophil – neutrophil count ($\times 10^9$/L); Monocyte – monocyte count ($\times 10^9$/L); Basophils – basophil count ($\times 10^9$/L); Eosinophils – eosinophil count ($\times 10^9$/L); HB – hemoglobin concentration (g/L); Hematocrit – volume percentage of red blood cells in blood (%); Platelet – platelet count ($\times 10^9$/L); ESR – erythrocyte sedimentation rate (mm/h); CRP – C-reactive protein (mg/L); CK – creatine kinase (U/L); CK_MB – creatine kinase-MB isoenzyme (U/L); TC – total cholesterol (mmol/L); TG – triglycerides (mmol/L); K – potassium (mmol/L); Na – sodium (mmol/L); Ca – calcium (mmol/L); Cl – chloride (mmol/L); Mg – magnesium (mmol/L); P – phosphorus/inorganic phosphate (mmol/L); Creatinine – serum creatinine (μmol/L); eGFR – estimated glomerular filtration rate (mL/min/1.73m^2); Uacid – uric acid (μmol/L); CYS_C – cystatin C (mg/L); HbA1c – glycated hemoglobin A1c (%); TP – total protein (g/L); ALB – albumin (g/L); AST – aspartate aminotransferase (U/L); ALT – alanine aminotransferase (U/L); AFU – alpha-L-fucosidase (U/L); NT_5 – 5'-nucleotidase (U/L); HDL – high-density lipoprotein cholesterol (mmol/L); LDL – low-density lipoprotein cholesterol (mmol/L); GGT – gamma-glutamyl transferase (U/L); ALP – alkaline phosphatase (U/L); LDH – lactate dehydrogenase (U/L); DBIL – direct (conjugated) bilirubin (μmol/L); IBIL – indirect (unconjugated) bilirubin (μmol/L); TBIL – total bilirubin (μmol/L); UMAlb – urinary microalbumin (mg/L); and UACR – urinary albumin-to-creatinine ratio (mg/g or mg/mmol). The white blood cell subtypes (lymphocytes, neutrophils, monocytes, eosinophils, basophils) are standard components of a complete blood count (CBC) differential.

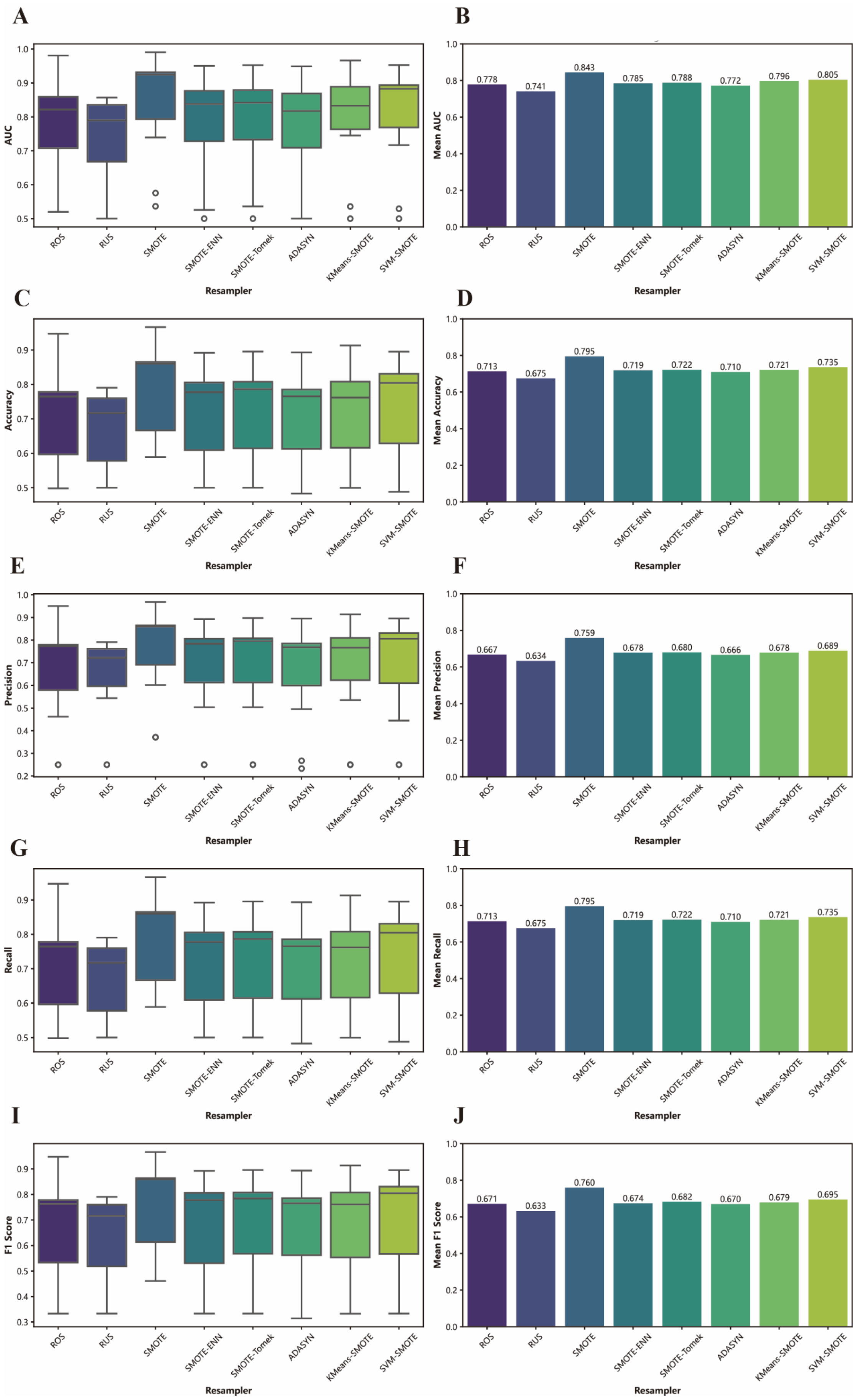

A
AUC
1.0
0.9
0.8
0.7
0.6
0.5
ROS
RUS
SMOTE
SMOTE-ENN
SMOTE-Tomek
ADASYN
KMeans-SMOTE
SVM-SMOTE
Resampler
B
Mean AUC
0.778
0.741
0.843
0.785
0.788
0.772
0.796
0.805
C
Accuracy
D
Mean Accuracy
0.713
0.675
0.795
0.719
0.722
0.710
0.721
0.735
E
Precision
F
Mean Precision
0.667
0.634
0.759
0.678
0.680
0.666
0.678
0.689
G
Recall
H
Mean Recall
0.713
0.675
0.795
0.719
0.722
0.710
0.721
0.735
I
F1 Score
J
Mean F1 Score
0.671
0.633
0.760
0.674
0.682
0.670
0.679
0.695

Supplementary Figure 1. Comparative Analysis of Different Resampling Techniques for Class Imbalance.

(A): Boxplots showing the distribution of Area Under the Curve (AUC) scores for each resampling method across cross-validation folds; (B): Bar chart displaying the mean AUC scores for each method; (C): Boxplots showing the distribution of accuracy scores; (D): Bar chart displaying the mean accuracy scores; (E): Boxplots showing the distribution of precision scores; (F): Bar chart displaying the mean precision scores; (G): Boxplots showing the distribution of recall scores; (H): Bar chart displaying the mean recall scores; (I): Boxplots showing the distribution of F1 scores; (J): Bar chart displaying the mean F1 scores. The techniques evaluated were Random Over-sampling (ROS), Random Under-sampling (RUS), Synthetic Minority Over-sampling TEchnique (SMOTE), SMOTE-ENN, SMOTE-Tomek, Adaptive Synthetic Sampling (ADASYN), KMeans-SMOTE, and SVM-SMOTE.

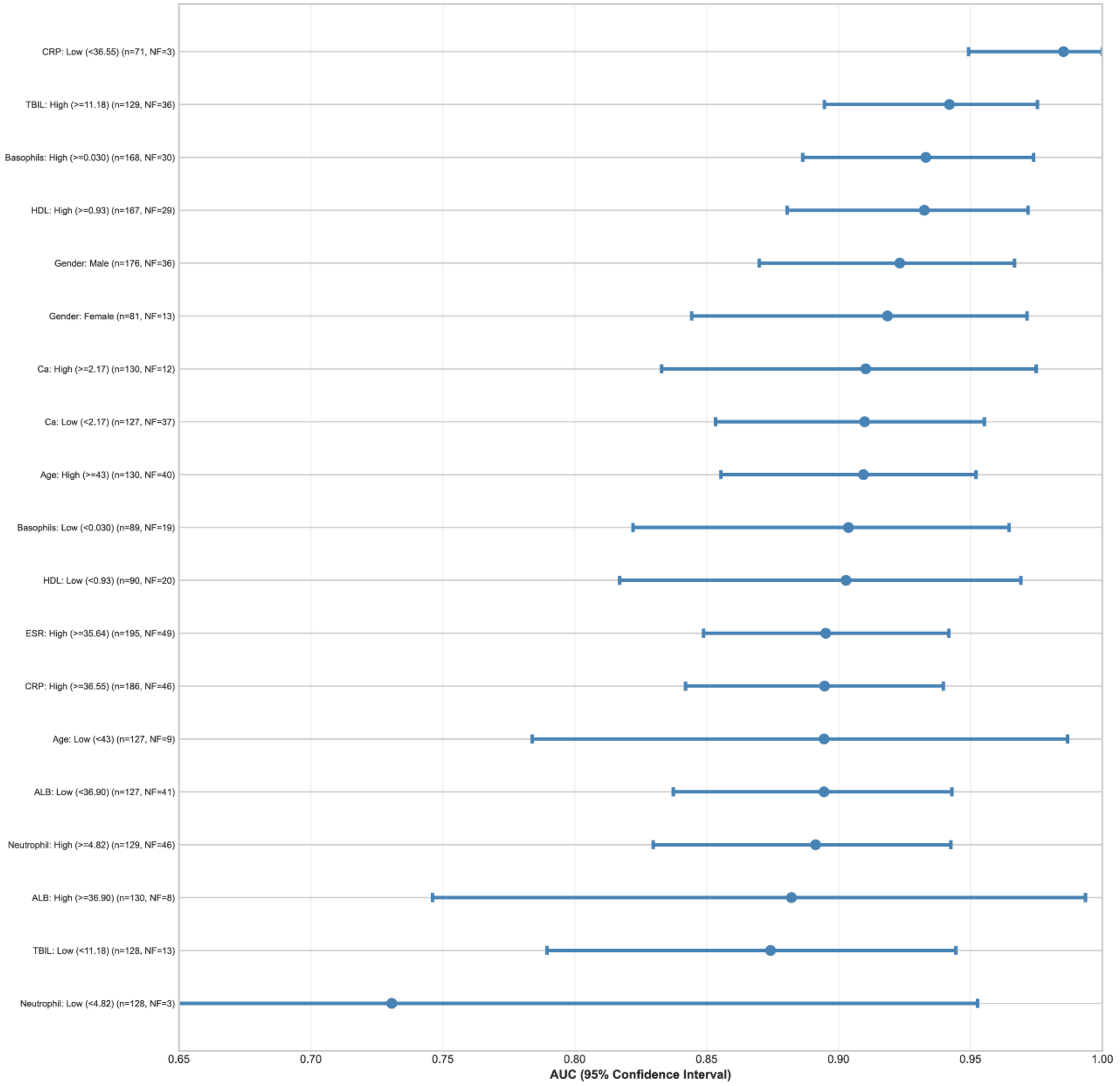

Supplementary Figure 2. Subgroup Analysis of Model Performance in the External Validation Cohort.

A forest plot displaying the Area Under the Curve (AUC) with 95% confidence intervals for the diagnostic model across various patient subgroups defined by clinical and laboratory parameters. The plot is organized with subgroups listed on the y-axis and the corresponding AUC value and its confidence interval represented horizontally on the x-axis. Each row corresponds to a specific subgroup, labeled with the defining variable (e.g., CRP, Neutrophil), its threshold (High/Low), the sample size (n), and the number of osteomyelitis cases (NF). A vertical reference line is included for visual comparison.

Supplementary Table 2 Subgroup Analysis of Model Using Selected Features

Feature	Subgroup	*N*	AUC	95%CI	Accuracy	F1	Precision	Recall
Age	Low (<43)	127.000	0.895	0.782-0.981	0.945	0.779	0.796	0.765
Age	High (>=43)	130.000	0.909	0.852-0.955	0.815	0.765	0.798	0.749
Gender	Female	81.000	0.919	0.845-0.969	0.852	0.684	0.722	0.663
Gender	Male	176.000	0.923	0.867-0.964	0.892	0.821	0.852	0.798
Neutrophil	Low (<4.82)	128.000	0.731	0.504-0.953	0.961	0.490	0.488	0.492
Neutrophil	High (>=4.82)	129.000	0.891	0.833-0.940	0.798	0.768	0.792	0.756
Basophils	Low (<0.030)	89.000	0.904	0.828-0.964	0.854	0.769	0.789	0.754
Basophils	High (>=0.030)	168.000	0.933	0.884-0.975	0.893	0.795	0.842	0.765
ESR	Low (<35.64)	62.000	-	-	-	-	-	-
ESR	High (>=35.64)	195.000	0.895	0.842-0.936	0.841	0.771	0.802	0.751
CRP	Low (<36.55)	71.000	0.985	0.943-1.000	0.958	0.689	0.736	0.659
CRP	High (>=36.55)	186.000	0.895	0.838-0.940	0.849	0.781	0.812	0.761
Ca	Low (<2.17)	127.000	0.910	0.852-0.959	0.858	0.823	0.835	0.812
Ca	High (>=2.17)	130.000	0.910	0.826-0.974	0.900	0.591	0.660	0.571
ALB	Low (<36.90)	127.000	0.894	0.837-0.945	0.795	0.756	0.770	0.747
ALB	High (>=36.90)	130.000	0.882	0.737-0.985	0.962	0.763	0.980	0.688
HDL	Low (<0.93)	90.000	0.903	0.832-0.967	0.844	0.766	0.778	0.757
HDL	High (>=0.93)	167.000	0.933	0.885-0.972	0.898	0.797	0.856	0.761
TBIL	Low (<11.18)	128.000	0.874	0.784-0.944	0.883	0.611	0.646	0.594
TBIL	High (>=11.18)	129.000	0.942	0.898-0.976	0.876	0.837	0.861	0.820

AUC (area under the receiver operating characteristic curve, indicating model discrimination), with 95% confidence intervals (95% CI) reported; F1 score, the harmonic mean of precision and recall; Precision (positive predictive value); Recall (sensitivity); ESR (erythrocyte sedimentation rate); CRP (C-reactive protein); Ca (calcium); ALB (albumin); HDL (high-density lipoprotein cholesterol); and TBIL (total bilirubin).

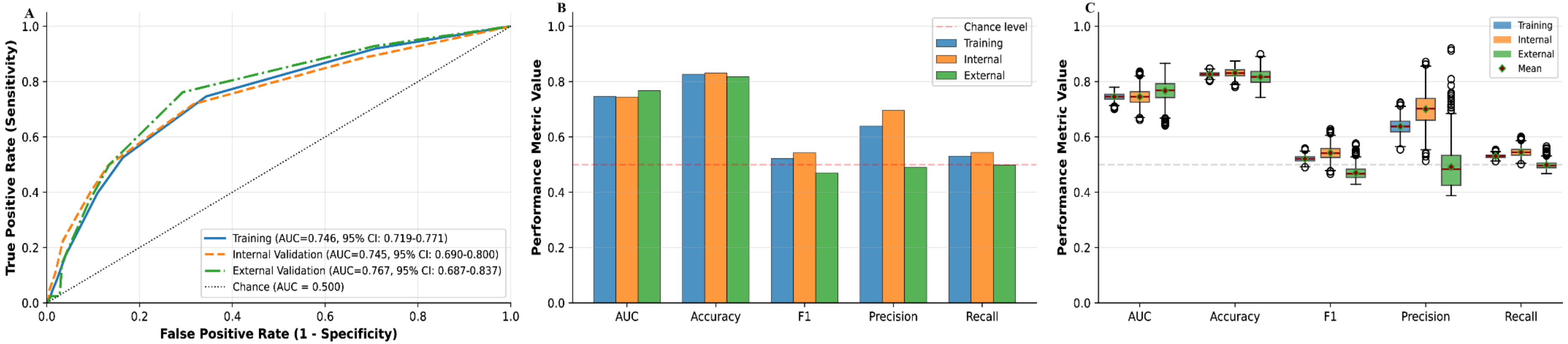

Supplementary Figure 3. Comprehensive performance and stability analysis of the LRINEC (Laboratory Risk Indicator for Necrotizing Fasciitis) score.

(A): Receiver operating characteristic (ROC) curves for the training, internal validation, and external validation cohorts, illustrating the trade-off between sensitivity and specificity with associated area under the curve (AUC) values and confidence intervals; (B): A grouped bar chart comparing five core diagnostic metrics—AUC, accuracy, F1-score, precision, and recall—across the three datasets to highlight performance variability relative to the theoretical chance level; (C): Box plots showing the distribution of performance metrics derived from 1,000 bootstrap iterations, with horizontal lines indicating the mean values to represent the statistical robustness and density of the results across all cohorts.

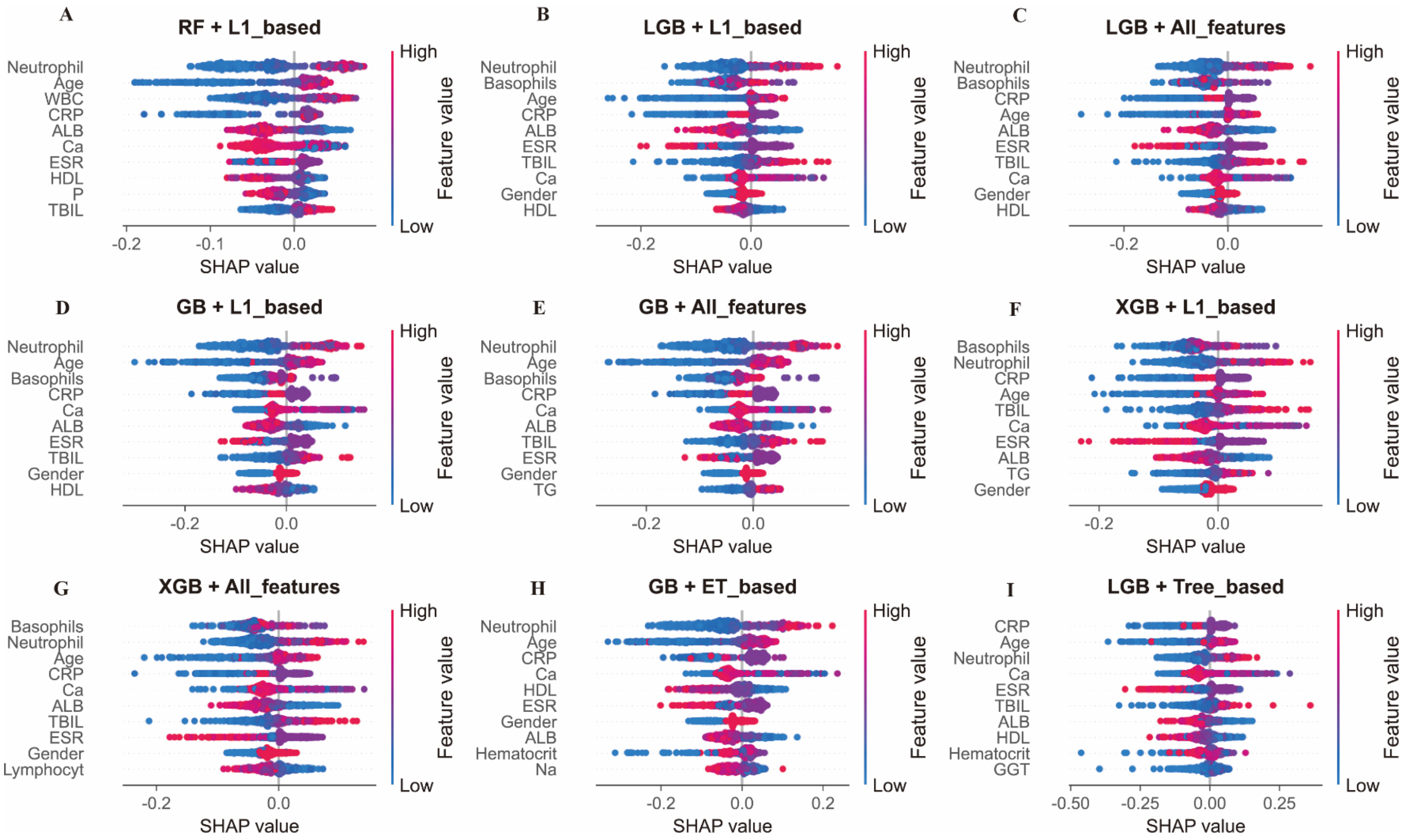

Supplementary Figure 4. SHAP Feature Importance Consistency Analysis Across Top Tuned Models.

(A): SHAP value distribution for Random Forest model with L1-based feature selection showing relative importance of different biomarkers; (B): SHAP value distribution for LightGBM model with L1-based feature selection highlighting feature importance ranking; (C): SHAP value patterns for LightGBM model utilizing all available clinical features; (D): Feature importance visualization for Gradient Boosting model with L1-based features demonstrating contribution magnitude of each biomarker; (E): Comprehensive SHAP analysis for Gradient Boosting model incorporating all clinical parameters; (F): XGBoost model performance with L1-based features showing differential impact of each laboratory marker; (G): SHAP value distribution for XGBoost model using all available clinical features; (H): Feature importance metrics for Gradient Boosting model with ET-based feature selection; (I): SHAP value visualization for LightGBM model using Tree-based feature selection techniques showing relative contribution of each biomarker to model predictions.

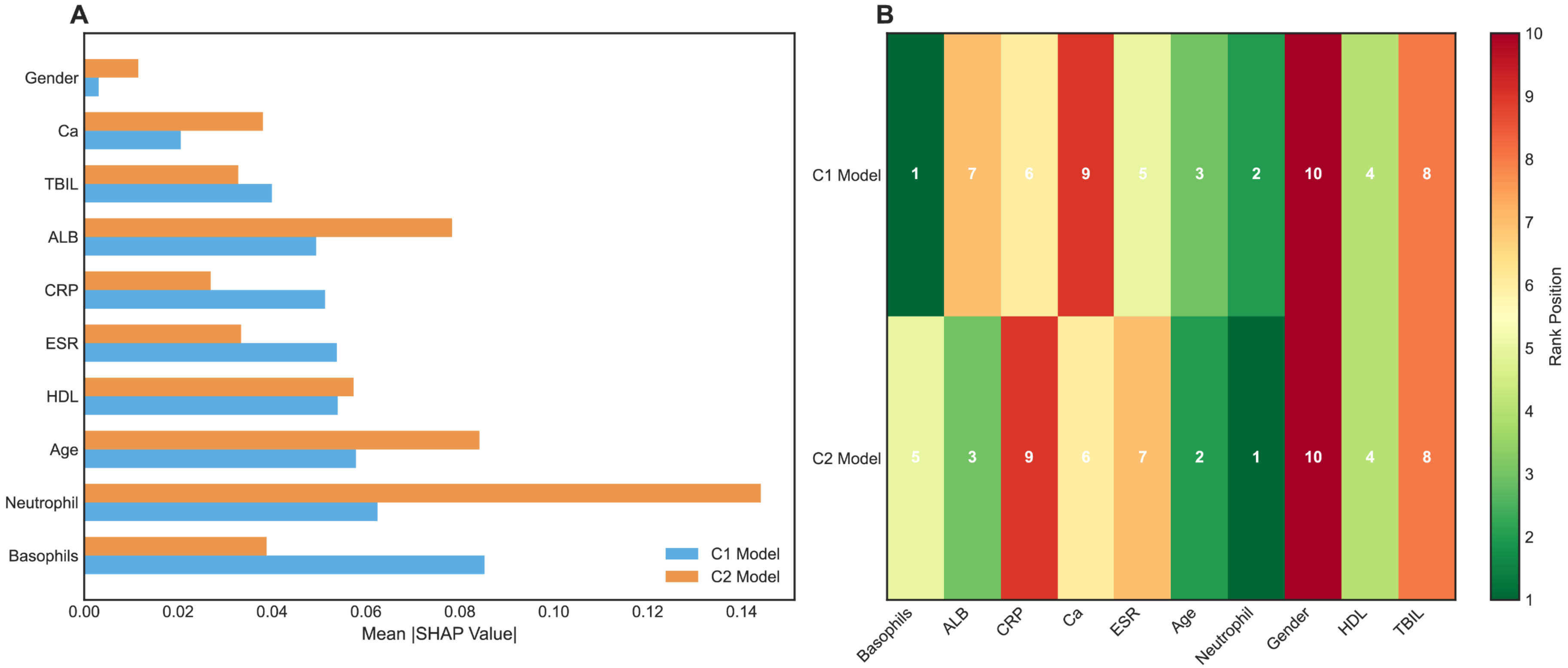

Supplementary Figure 5. Cross-Center SHAP Feature Importance Comparison.

(A): Side-by-side bar plots showing mean absolute SHAP values for all 10 features. Blue bars represent Center 1 model; orange bars represent Center 2 model. Both models identify Neutrophil and Age among top features, demonstrating core consistency. (B): Rank position heatmap visualizing feature importance rankings across both models. Color intensity indicates rank position (1= green, 10= red).

Supplementary Table 3 Comparison of Top Feature Rankings by SHAP Values Between Center 1 and Center 2 Models

Feature	C1_Rank	C1_SHAP	C2_Rank	C2_SHAP	Rank Difference	Category
Basophils	1	0.085255329	5	0.038846506	4	Inflammatory
Neutrophil	2	0.062429713	1	0.144085513	1	Inflammatory
Age	3	0.057860067	2	0.084166298	1	Demographic
HDL	4	0.053971894	4	0.057374189	0	Metabolic
ESR	5	0.053795461	7	0.033391836	2	Inflammatory
CRP	6	0.051297104	9	0.026926241	3	Inflammatory
ALB	7	0.049368373	3	0.07833853	4	Metabolic
TBIL	8	0.039967467	8	0.032801917	0	Metabolic
Ca	9	0.020539713	6	0.0380505	3	Metabolic
Gender	10	0.003087422	10	0.011476161	0	Demographic

Abbreviations: C1, Center 1; C2, Center 2; SHAP, SHapley Additive exPlanations; HDL, High-Density Lipoprotein; ESR, Erythrocyte Sedimentation Rate; CRP, C-Reactive Protein; ALB, Albumin; TBIL, Total Bilirubin; Ca, Calcium. Rank Difference was calculated as the absolute difference between C1_Rank and C2_Rank. Features were categorized based on clinical domain (Inflammatory, Metabolic, or Demographic). Spearman rank correlation analysis demonstrated strong consistency in feature importance rankings between the two centers ($\rho = 0.66$, $P = 0.04$), with core inflammatory markers (Neutrophil, ESR, CRP) maintaining top-nine positions in both models, supporting cross-center interpretability stability rather than center-specific dependencies.
